# CUL4B promotes hepatocellular carcinoma progression and oxaliplatin resistance by facilitating FUS degradation

**DOI:** 10.1038/s41419-025-08320-6

**Published:** 2025-12-14

**Authors:** Jun-Dong Wei, Kai Cheng, Xiaoyu Wu, Luyi Zhang, Qinle Wang, Yetian Liang, Ziqi Zhu, Weijia Meng, Wangyang Chen, Xingang Guan, Hui Yang, Lisha Zhou

**Affiliations:** 1 Laboratory of Tumor Biology and Translational Research, School of Pharmaceutical Sciences, Taizhou University, Taizhou, Zhejiang China; 2 Department of Basic Medicine, School of Medicine, Taizhou University, Taizhou, Zhejiang China; 3 Taizhou Central Hospital (Taizhou University Hospital), Taizhou University, Taizhou, Zhejiang China; 4 Department of Neurosurgery, Huashan Hospital, Fudan University, Shanghai, China

**Keywords:** Oncogenes, Ubiquitylation, Neddylation

## Abstract

Cullin4B (CUL4B), which functions as a scaffold protein within the CUL4B-RING ubiquitin ligase complex (CRL4B), is frequently overexpressed in various cancers and exhibits oncogenic characteristics. However, its specific role in hepatocellular carcinoma (HCC) progression and drug resistance remains unclear. This study revealed that CUL4B is upregulated in HCC tissues, with elevated levels correlating with poor patient prognosis. A strong inverse relationship was observed between the expression of CUL4B and the tumor suppressor miR-143-3p in human HCC samples; CUL4B knockdown significantly increased miR-143-3p levels. Moreover, suppression of CUL4B inhibited HCC cell proliferation and enhanced sensitivity to oxaliplatin through miR-143-3p upregulation. Fused in sarcoma (FUS), an RNA-binding protein implicated in miRNA biogenesis, was identified as a novel CRL4B^DTL^ substrate. CUL4B facilitates FUS ubiquitination and subsequent degradation, thereby reducing FUS protein levels. This reduction impairs miR-143-3p formation, activates the KRAS signaling pathway, and promotes tumor progression and oxaliplatin resistance. In summary, this study provided compelling evidence that CUL4B knockdown may be a promising strategy for treating HCC and increasing tumor cell sensitivity to oxaliplatin therapy.

## Introduction

Hepatocellular carcinoma (HCC) ranks as the most common primary liver malignancy and the third leading cause of cancer-related deaths worldwide [1]. The majority of HCC patients are diagnosed at intermediate or advanced stages, characterized by high malignancy, frequent recurrence, and poor prognosis, posing a severe threat to patient survival [2]. Oxaliplatin-based chemotherapy remains a cornerstone treatment for unresectable HCC, significantly improving survival in selected patient cohorts [3, 4]. However, its efficacy is substantially limited by intrinsic or acquired resistance, which occurs in a high proportion of HCC cases [5]. Therefore, elucidating the molecular drivers of HCC progression and oxaliplatin resistance is urgently needed to identify novel therapeutic targets and improve patient outcomes.

Neddylation is a post-translational modification that involves the attachment of a ubiquitin-like peptide, NEDD8, to a lysine residue on the substrate protein. This process is facilitated by a cascade of enzymes, including NEDD8-activating enzyme E1 (NAE), NEDD8-conjugating enzyme E2 (UBE2M/UBE2F), and several neddylation ligases E3 [6–8]. This modification critically regulates protein stability, activity, and complex assembly [9, 10]. Hyperactivation of the neddylation pathway, including overexpression of NEDD8 and its associated enzymes, is frequently observed in multiple human cancers and correlates with poor prognosis [11–13]. Consequently, targeting neddylation has emerged as a promising anticancer strategy. MLN4924 (pevonedistat), a first-in-class small-molecule inhibitor of NAE [14], is currently undergoing phase I/II clinical trials, both as a monotherapy drug and in combination with other chemotherapeutic agents [15–20]. The best-characterized neddylation substrates are the cullin family members (CUL1, 2, 3, 4 A, 4B, and 5), which serve as molecular scaffolds for Cullin–RING E3 ubiquitin ligases (CRLs) [6]. Neddylation induces conformational changes in cullins, facilitating CRL activation and subsequent substrate ubiquitination [21, 22]. As the largest E3 ubiquitin ligase family, CRLs regulate diverse cellular processes by targeting specific proteins for degradation or functional modulation [23].

CUL4B, in particular, assembles with an adapter protein (DDB1), a RING-finger component (RBX1), and various substrate receptors known as DDB1-and CUL4-associated factors (DCAFs) to form various CRL4B E3 ubiquitin ligase complexes [24]. CRL4B mediates many important processes, either by catalyzing the polyubiquitination of proteins for proteasomal degradation or mono-ubiquitination of H2A for epigenetic modifications [25–30]. CUL4B is overexpressed in multiple cancers, including breast [26], prostate [31], and bladder cancers [32], and is closely associated with tumor development and prognosis. However, its functional role and regulatory mechanisms in HCC, especially in the context of chemotherapy resistance, remain incompletely understood.

microRNAs (miRNAs) are a class of non-coding RNA molecules that play a crucial role in regulating cell differentiation, proliferation, survival, and resistance to anticancer therapy. They perform this role by binding to complementary target mRNAs, which results in either translational inhibition or degradation [33, 34]. Due to their ability to simultaneously modulate multiple genes within signaling networks, miRNAs have emerged as critical players in tumor biology [35, 36]. Numerous miRNAs are aberrantly expressed in human cancers, and several miRNA-based therapeutics have progressed to clinical trials for cancer treatment (ClinicalTrials.gov: NCT04811898, NCT03010722, NCT01829971, etc.) [37–40]. Of particular interest is miR-143-3p, which is a tumor suppressor and an independent prognostic marker in various cancers [41–44]. The tumor-suppressive effects of miR-143-3p are largely attributed to its direct targeting of well-known oncogenes such as *KRAS* [44–46]. Nevertheless, the upstream regulatory mechanisms controlling miR-143-3p expression and its functional role in oxaliplatin resistance in HCC are not fully defined.

In this study, we investigated the role of CUL4B in HCC progression and oxaliplatin resistance, with a focus on its regulation of miR-143-3p. We identified a strong inverse correlation between CUL4B and miR-143-3p in HCC tissues, with their combined expression serving as a prognostic biomarker. Mechanistically, we demonstrated that CUL4B, in complex with DTL, promotes the ubiquitination and degradation of the RNA-binding protein FUS, a known facilitator of miRNA biogenesis. This in turn suppresses miR-143-3p maturation, leading to activation of the KRAS signaling pathway and ultimately driving tumor growth and oxaliplatin resistance. Our findings reveal a previously unrecognized CUL4B/FUS/miR-143-3p/KRAS regulatory axis and highlight CUL4B as a potential therapeutic target for HCC treatment.

## Results

### CUL4B inversely correlates with miR-143-3p, and high CUL4B/low miR-143-3p levels are associated with poor survival of patients with HCC

**Fig. 1:**
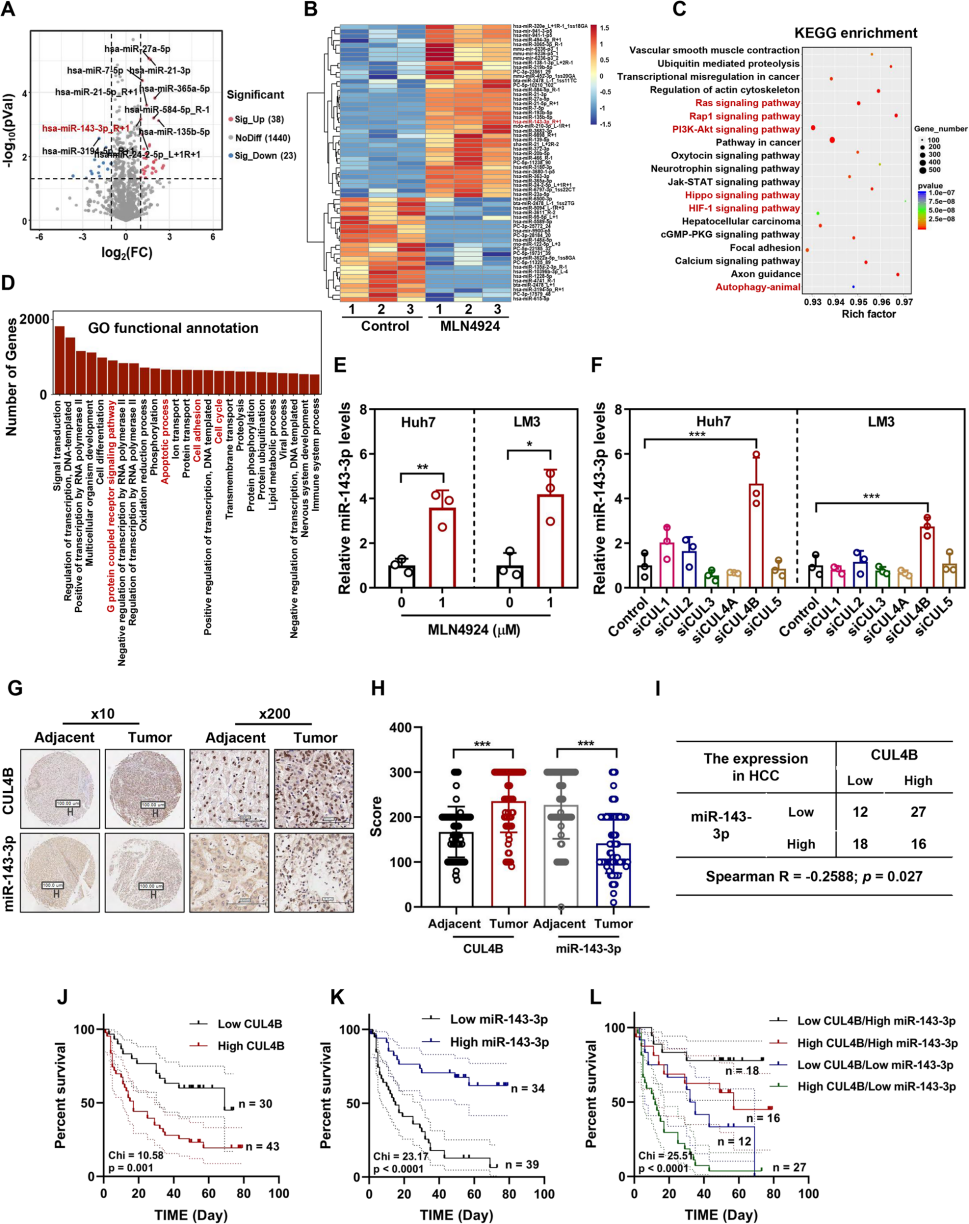
CUL4B inversely correlates with miR-143-3p, and high CUL4B/low miR-143-3p expression is associated with poor survival of patients with HCC. **A** Volcano plot showing miRNA expression profile after MLN4924 treatment. Conditions for screening differences: │Fold Change│ > 2; *p* < 0.05. The red and blue points in the plot indicate significantly upregulated and downregulated miRNAs, respectively. **B** Hierarchical clustering of differentially expressed miRNAs in two groups. **C** KEGG pathway analysis of differentially expressed miRNAs. **D** GO function analysis of differentially expressed miRNAs. **E** Level of miR-143-3p expression determined using qRT-PCR following 24 h treatment of Huh7 and LM3 cells with 1 μΜ MLN4924 (mean ± sD, *n* = 3). **F** Level of miR-143-3p expression determined using qRT-PCR following downregulation of CULs in Huh7 and LM3 cells by siRNA interference for 72 h (mean ± SD, *n* = 3). **G** Images of CUL4B and miR-143-3p in HCC and normal tissues (× 10, scale bar: 100 μm; × 200, scale bar: 100 μm). IHC staining of human HCC tissue arrays was performed using a specific antibody against CUL4B. ISH staining of HCC tissue arrays was conducted with a specific probe targeting miR-143-3p. Adjacent, *n* = 69; Tumor, *n* = 73. **H** Statistical analysis of IHC (CUL4B) and ISH scores (miR-143-3p) for HCC and normal tissues (mean ± SD). Tissue sections were semi-quantitatively assessed based on the percentage of positively stained tumor cells and the intensity of staining. **I** Correlation analysis of CUL4B and miR-143-3p expression scores for HCC tissues. The Spearman non-parametric correlation test was used to assess association. **J** Overall survival analysis of 73 patients with HCC based on CUL4B IHC scores. The log-rank test was used to estimate significance. **K** Overall survival analysis of 73 patients with HCC based on miR-143-3p ISH scores. The log-rank test was used to estimate significance. **L** Overall survival analysis of 73 patients with HCC based on combined CUL4B IHC scores and miR-143-3p ISH scores. The log-rank test was used to estimate significance. Two-tailed, unpaired *t*-test was used for (**E**, **H**). One-way ANOVA/LSD test was used for (**F**). Data are presented as the mean ± standard deviation derived from a minimum of three independent experiments. Statistical significance was assessed using the appropriate testing methods, with *p < 0.05, **p < 0.01, and ***p < 0.001 denoting levels of significance.

To investigate the effect of neddylation-activated CRLs on miRNA expression in HCC, Huh7 cells were subjected to treatment with or without 1 μM MLN4924 for 24 h, followed by miRNA sequencing. The treatment significantly altered the expression of 61 miRNAs (38 upregulated and 23 downregulated), compared to that in the control group (｜Fold Change｜ > 2.0 and *p* < 0.05) (Fig. 1A, B). ‌Gene Ontology (GO) functional annotation and Kyoto Encyclopedia of Genes and Genomes (KEGG) enrichment analysis indicated that the target genes of these miRNAs were primarily involved in key signaling pathways related to HCC progression, apoptosis, and the cell cycle (Fig. 1C, D). Among the differentially expressed miRNAs, miR-143-3p is recognized as a tumor suppressor; low expression levels of this miRNA are linked to poor overall survival in several cancers [41–44]. The RNA sequencing results were validated by assessing miR-143-3p expression in two HCC cell lines. Inhibition of neddylation-activated CRLs by MLN4924 significantly elevated miR-143-3p levels in both Huh7 and LM3 cells (Fig. 1E).

The inhibition of neddylation by MLN4924 is likely achieved through the inactivation of one or more downstream CRLs. To identify the specific CRL involved, each cullin gene was individually silenced in Huh7 and LM3 cells using small interfering RNA (siRNA). Only CUL4B knockdown increased miR-143-3p expression (Fig. 1F). Further analysis of CUL4B and miR-143-3p expression in 73 HCC tissues and 69 adjacent normal tissues using immunohistochemistry (IHC) and in situ hybridization (ISH) revealed a marked upregulation of CUL4B and a corresponding downregulation of miR-143-3p in HCC tissues (Fig. 1G, H). Spearman’s correlation analysis confirmed a significant negative correlation between CUL4B and miR-143-3p expression in HCC tissues (Fig. 1I).

Clinical data were correlated with CUL4B and miR-143-3p expression levels in HCC samples (Supplemental Table 1). Survival receiver operating characteristic (ROC) curve analysis was conducted based on the CUL4B and miR-143-3p expression scores, and the respective Youden index cutoffs were determined (CUL4B, AUC = 0.661; miR-143-3p, AUC = 0.203) (Supplemental Fig. 1A, B). Patients were categorized into low (*n* = 30) and high (*n* = 43) CUL4B expression groups based on the cutoff values and similarly into low (*n* = 34) and high (*n* = 39) miR-143-3p expression groups. High CUL4B or low miR-143-3p expression was associated with poor overall survival in both univariate (CUL4B: *p* = 0.002, HR = 2.755; miR-143-3p: *p* < 0.001, HR = 0.225) and multivariate analyses (CUL4B: *p* = 0.046, HR = 1.980; miR-143-3p: *p* < 0.001, HR = 0.257) (Supplemental Table 2), suggesting that CUL4B and miR-143-3p may serve as independent prognostic indicators for patients with HCC. Kaplan–Meier analysis demonstrated that patients with HCC exhibiting high CUL4B or low miR-143-3p expression had significantly lower overall survival rates (CUL4B, *p* = 0.001; miR-143-3p, *p* < 0.001; log-rank test) (Fig. 1J, K). Patients with high CUL4B and low miR-143-3p expression had the poorest overall prognosis (Fig. 1L). These findings emphasize that CUL4B expression inversely correlates with that of miR-143-3p and that high CUL4B/low miR-143-3p expression serves as a predictor of poor HCC prognosis, suggesting that the *CUL4B*/miR-143-3p axis may constitute an oncogene–tumor suppressor cascade that drives HCC progression.

### CUL4B knockdown inhibits the growth of HCC cells and increases their susceptibility to oxaliplatin

**Fig. 2:**
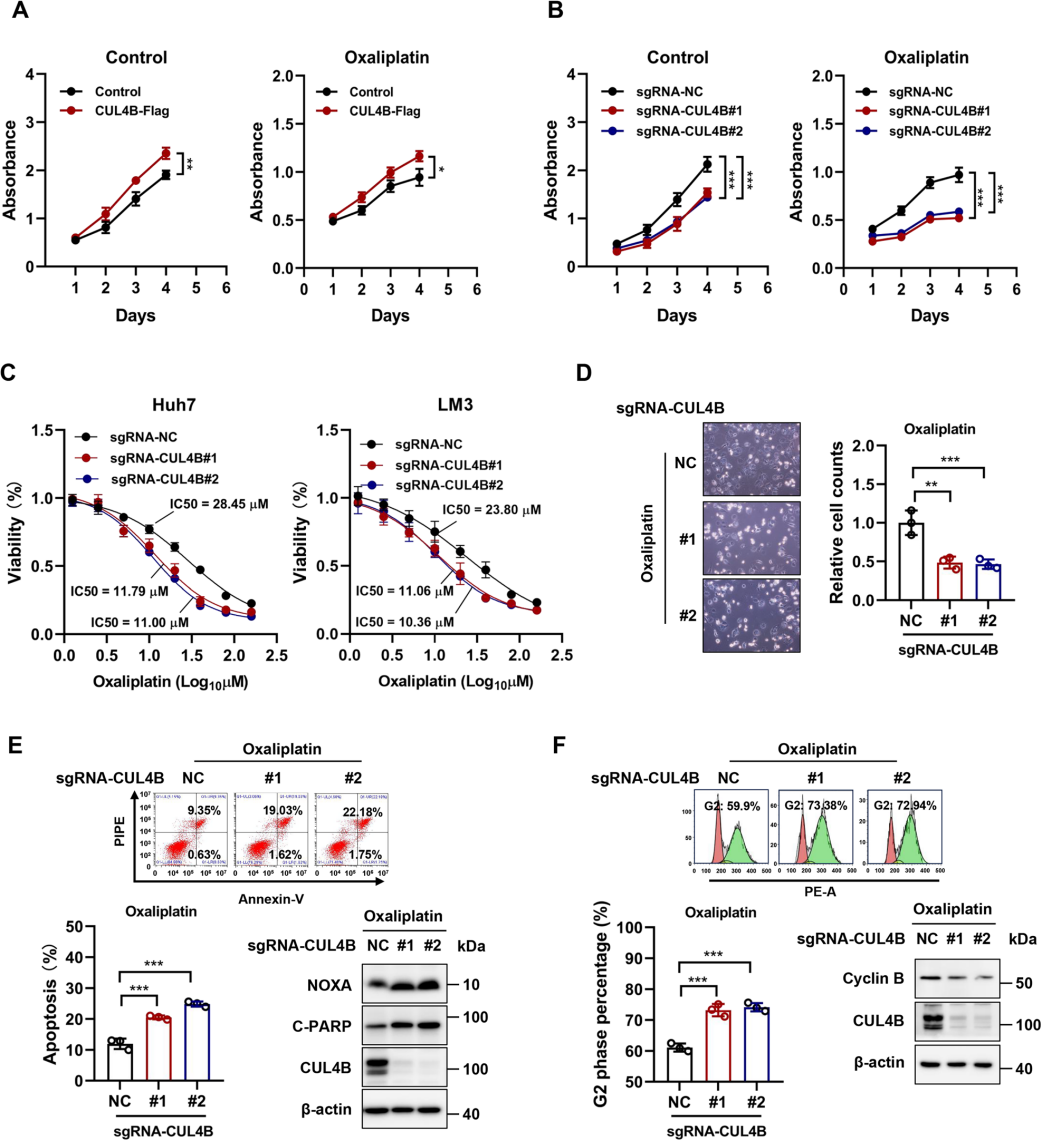
CUL4B knockdown inhibits the growth of HCC cells and increases their susceptibility to oxaliplatin. **A**, **B** Effect of CUL4B overexpression (**A**) or knockdown (**B**) on the proliferation of Huh7 cells treated with or without 2 μM oxaliplatin. Cell proliferation of HCC cells was determined using a CCK8 growth assay for 4 days (mean ± SD, *n* = 3). **C** Effect of CUL4B knockdown on IC_50_ of oxaliplatin in Huh7 and LM3 cells. IC_50_ was determined using a ATP-lite assay on cells treated with oxaliplatin at indicated concentrations for 48 h (mean ± SD, *n* = 6). **D** Effect of CUL4B knockdown on cellular morphology of Huh7 cells treated with 40 μΜ oxaliplatin for 48 h. Cell number was counted (mean ± SD, *n* = 3). **E** Effect of CUL4B knockdown on apoptosis of Huh7 cells treated with 40 μΜ oxaliplatin for 48 h. Annexin V-FITC/PI double-staining analysis was used to analyze cell apoptosis (mean ± SD, *n* = 3). Western blotting was used to assess the expression of the apoptosis-related proteins NOXA and cleaved-PARP. **F** Effect of CUL4B knockdown on the cell cycle of Huh7 cells treated with 40 μΜ oxaliplatin for 48 h. PI staining and FACS analysis were used to analyze the cell cycle profile (mean ± SD, *n* = 3). Western blotting was used to assess the expression of the cell cycle-related protein Cyclin B. Two-tailed, unpaired *t*-test was used for (**A**). One-way ANOVA/LSD test was used for (**B**−**F**). Data are presented as the mean ± standard deviation derived from a minimum of three independent experiments. Statistical significance was assessed using the appropriate testing methods, with *p < 0.05, **p < 0.01, and ***p < 0.001 denoting levels of significance.

To elucidate the fundamental role of CUL4B in regulating the growth and survival of human HCC cells, gain- and loss-of-function analyses were conducted *via* CUL4B overexpression and knockdown in Huh7 cells. The efficacy of CUL4B manipulation was confirmed using western blotting (Supplemental Fig. 2A, B). Cell Counting Kit-8 (CCK8) assay demonstrated that CUL4B overexpression significantly promoted cell proliferation, whereas its knockdown inhibited cell growth, underscoring the involvement of CUL4B in HCC progression (Fig. 2A, B). Additionally, CUL4B overexpression protected Huh7 cells from oxaliplatin-mediated growth suppression, whereas CUL4B knockdown amplified the effect of this drug (Fig. 2A, B). ATP-lite assay revealed that CUL4B knockdown reduced the IC_50_ of oxaliplatin in both Huh7 and LM3 cells (Fig. 2C).

Oxaliplatin induces cell cycle arrest and apoptosis, thus significantly reducing tumor cell viability [47, 48]. CUL4B knockdown increased cell death following oxaliplatin treatment (Fig. 2D). CUL4B knockdown sensitized Huh7 cells to oxaliplatin-induced apoptosis, as evidenced by a significant increase in Annexin V-positive cells (detected *via* FACS analysis) and the accumulation of the apoptosis-related proteins, NOXA and cleaved PARP (c-PARP) (Fig. 2E). Furthermore, cell cycle analysis demonstrated a significant increase in the proportion of cells in the G2 phase among CUL4B-knockdown cells treated with oxaliplatin, compared to control cells. This finding was further supported by the downregulation of Cyclin B expression, which is one of the hallmarks of G2 phase arrest (Fig. 2F). These results suggest that CUL4B knockdown suppressed the growth of HCC cells and increased their susceptibility to oxaliplatin by inducing cell cycle arrest and apoptosis. Hence, CUL4B may potentially drive HCC growth and oxaliplatin resistance.

### CUL4B knockdown enhances the sensitivity of HCC cells to oxaliplatin by upregulating miR-143-3p expression

**Fig. 3:**
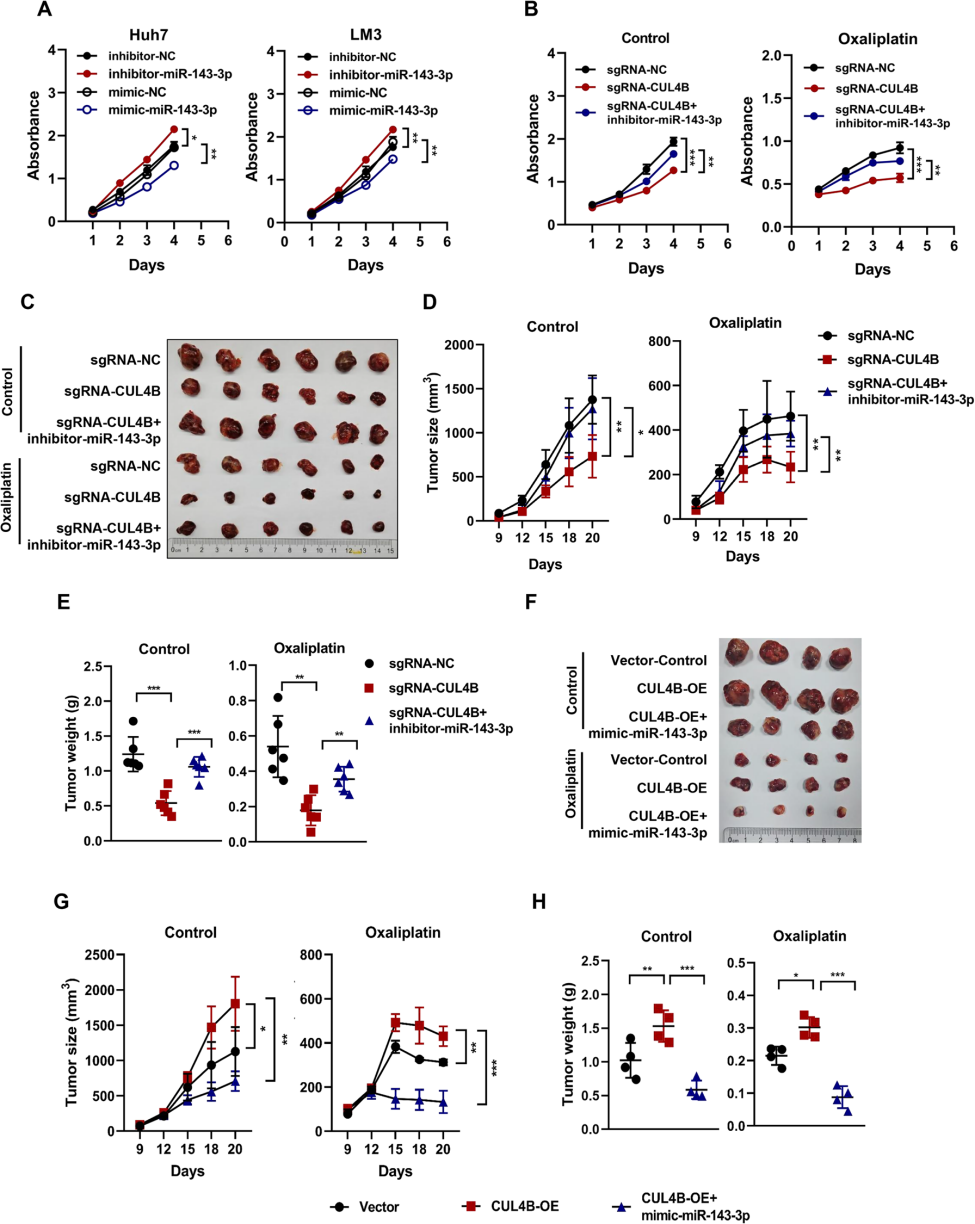
CUL4B knockdown enhances the sensitivity of HCC cells to oxaliplatin by upregulating miR-143-3p expression. **A** Effect of miR-143-3p overexpression or inhibition on the proliferation of Huh7 and LM3 cells. Cells were transfected with mimics or inhibitors of miR-143-3p and their respective negative controls. Cell proliferation of HCC cells was measured using a CCK8 growth assay for 4 days (mean ± SD, *n* = 3). **B** Effect of miR-143-3p inhibition on cell proliferation of CUL4B-knockdown Huh7 cells treated with or without 2 μM oxaliplatin. Cell proliferation of HCC cells was measured using a CCK8 growth assay for 4 days (mean ± SD, *n* = 3). **C**−**E** Inhibition of miR-143-3p significantly reversed the tumor growth inhibition and oxaliplatin sensitivity induced by CUL4B knockdown. Tumor tissues harvested and photographed on the day of sacrifice (*n* = 6 per group) (**C**). Tumor size measured with calipers at the indicated time points and converted to a tumor growth curve (**D**). Tumor weights of each group at the experiment endpoint (**E**). **F**−**H** Overexpression of miR-143-3p significantly blocked the tumor growth and oxaliplatin resistance caused by CUL4B overexpression. Tumor tissues harvested and photographed on the day of sacrifice (*n* = 4 per group) (**F**). Tumor size measured with calipers at the indicated time points and converted to a tumor growth curve (**G**). Tumor weights of each group at the experiment endpoint (**H**). Two-tailed, unpaired *t*-test was used for (**A**). Two-way ANOVA/LSD test was used for (**B**，**D, E, G, H**). Data are presented as the mean ± standard deviation derived from a minimum of three independent experiments. Statistical significance was assessed using the appropriate testing methods, with *p < 0.05, **p < 0.01, and ***p < 0.001 denoting levels of significance.

miR-143-3p suppresses various types of tumors [41–44]. To further investigate its role in HCC, Huh7 and LM3 cells were transfected with mimics or inhibitors of miR-143-3p and their respective negative controls. Overexpression of miR-143-3p significantly inhibited cell growth, whereas its inhibition promoted the proliferation of both Huh7 and LM3 cells (Fig. 3A). These findings reinforce the notion that miR-143-3p exerts a growth-suppressive effect on HCC. Given the inverse correlation between miR-143-3p and CUL4B, we explored whether miR-143-3p contributes to the suppression of HCC growth and increased sensitivity to oxaliplatin observed after CUL4B knockdown. Transfection of CUL4B-knockdown cells, which exhibited elevated miR-143-3p levels, with miR-143-3p inhibitors partially reversed the growth-suppressive effects of CUL4B knockdown both in the presence and absence of oxaliplatin (Fig. 3B).

A mouse xenograft tumor model was used to confirm whether these in vitro effects could be replicated in vivo. Tumor-bearing mice implanted with CUL4B-knockdown or wild-type Huh7 cells were treated with a vehicle or oxaliplatin. Additionally, antagomiR-143-3p, an in vivo-suitable miR-143-3p inhibitor, was intratumorally injected into CUL4B-knockdown tumors (Supplemental Fig. 3A). CUL4B knockdown significantly inhibited tumor growth, as evidenced by a reduction in tumor size and weight. However, miR-143-3p inhibition significantly reversed the tumor growth suppression induced by CUL4B knockdown (Fig. 3C–E). Similarly, CUL4B knockdown greatly enhanced the sensitivity of Huh7 cells to oxaliplatin treatment, whereas miR-143-3p inhibition repaired the growth defects observed in CUL4B knockdown cells (Fig. 3C–E). Subsequently, we administered agomiR-143-3p, a suitable mimic for in vivo studies, into tumors that overexpress CUL4B (Supplemental Fig. 3B). Our findings indicate that the overexpression of CUL4B indeed promotes tumor growth while reducing sensitivity to oxaliplatin (Fig. 3F–H). Furthermore, the introduction of miR-143-3p effectively mitigated the oxaliplatin resistance associated with CUL4B overexpression (Fig. 3F–H). These results are consistent with our in vitro findings and collectively suggest that inhibition of miR-143-3p is partially necessary for CUL4B-mediated tumor growth and resistance to oxaliplatin.

### CUL4B inhibits miR-143-3p expression by ubiquitinating and degrading FUS

**Fig. 4:**
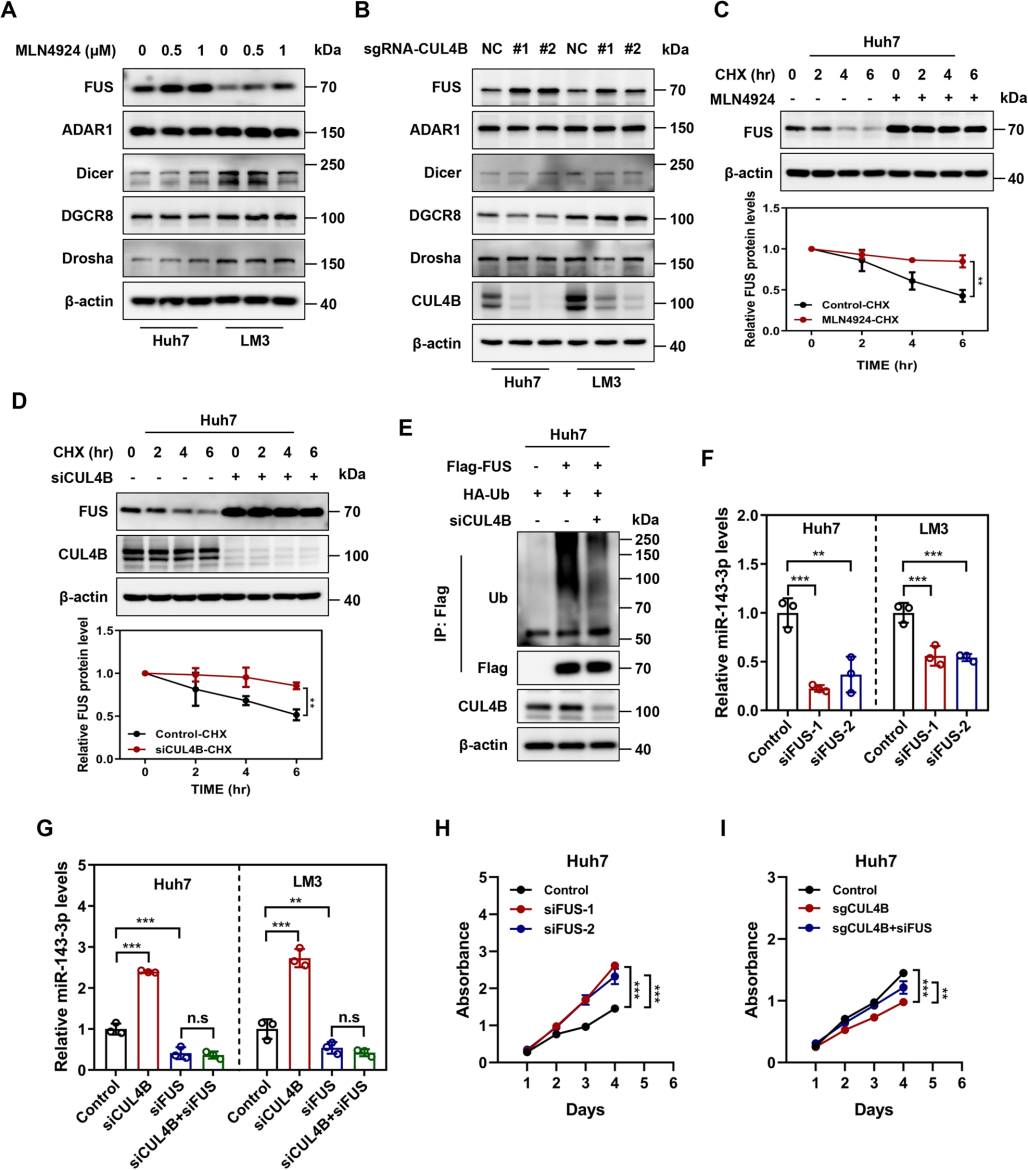
CUL4B inhibits miR-143-3p expression mediated by FUS. **A** FUS protein level determined using western blotting after treating Huh7 and LM3 cells with MLN4924 at indicated concentrations for 24 h. **B** FUS protein level determined using western blotting after CUL4B knockdown in Huh7 and LM3 cells, with quantification normalized to β-actin loading control. **C** Half-life of FUS in MLN4924-treated Huh7 cells. Huh7 cells were treated with 50 μg/mL CHX along with DMSO or MLN4924 (1 μM) and then harvested for western blotting analysis. FUS protein expression was quantified using ImageQuant TL software, with levels normalized to that of β-actin for comparison (mean ± SD, *n* = 3). **D** Half-life of FUS in CUL4B-knockdown cells. Huh7 cells were transfected with control or CUL4B siRNA for 72 h and then treated with 50 μg/mL CHX at the indicated time points before being subjected to western blotting analysis. FUS protein expression was quantified using ImageQuant TL software, with levels normalized to that of β-actin for comparison (mean ± SD, *n* = 3). **E** Effect of CUL4B on FUS ubiquitination. Vectors encoding Flag-tagged FUS and HA–Ub were transfected into CUL4B-knockdown Huh7 cells for 48 h, as indicated. All cells were treated with 10 μM MG132 for 6 h before being subjected to immunoprecipitation with anti-Flag M2 affinity resin and western blotting analysis. **F** Level of miR-143-3p expression determined using qRT-PCR following FUS downregulation in Huh7 and LM3 cells by siRNA interference for 72 h (mean ± SD, *n* = 3). **G** Effect of FUS on miR-143-3p expression level in CUL4B-knockdown Huh7 and LM3 cells. CUL4B-knockdown Huh7 and LM3 cells were transfected with control or FUS siRNA for 72 h and then harvested for qRT-PCR (mean ± SD, *n* = 3). **H** Effect of FUS on the proliferation of Huh7 cells. Huh7 cells were transfected with control or FUS siRNA. Cell proliferation was measured using a CCK8 growth assay for 4 days (mean ± SD, *n* = 3). **I** Effect of FUS on the proliferation of CUL4B-knockdown Huh7 cells. CUL4B-knockdown Huh7 cells were transfected with control or FUS siRNA. Cell proliferation was measured using a CCK8 growth assay for 4 days (mean ± SD, *n* = 3). Two-tailed, unpaired *t*-test was used for (**C**, **D**) One-way ANOVA/LSD test was used for (**F**, **H**). Two-way ANOVA/LSD test was used for (**G**, **I**). Data are presented as the mean ± standard deviation derived from a minimum of three independent experiments. Statistical significance was assessed using the appropriate testing methods, with **p < 0.01, and ***p < 0.001 denoting levels of significance.

The abundance of miRNAs depends on the levels of microprocessor components. The effects of MLN4924 treatment and CUL4B knockdown were examined to assess the impact of CUL4B on microprocessor components, including Drosha, DGCR8, Dicer, QKI, ADAR1, and FUS. Both interventions led to an accumulation of FUS in Huh7 and LM3 cells (Fig. 4A, B), inspiring the hypothesis that FUS may serve as a target of CRL4B E3 ubiquitin ligase in HCC. To test this hypothesis, protein half-life assays were conducted to determine whether CRL4B E3 ligase regulates FUS stability. Cycloheximide (CHX) was used to inhibit protein translation, revealing that FUS is unstable (Supplemental Fig. 4A). Additionally, treatment with the proteasome inhibitor MG132 led to the accumulation of FUS and extended its half-life (Supplemental Fig. 4B, C). Furthermore, inhibition of all CRL activity *via* MLN4924 treatment or CUL4B knockdown alone prolonged the half-life of FUS (Fig. 4C, D). To explore the role of CUL4B in FUS regulation and assess the influence of other cullins on FUS expression, each cullin gene was individually silenced in Huh7 cells. Only CUL4B knockdown led to FUS accumulation (Supplemental Fig. 4D). An in vivo ubiquitination assay revealed that FUS was subject to polyubiquitination, a process that was inhibited by CUL4B knockdown (Fig. 4E). Collectively, our ubiquitination assays and protein stability experiments demonstrate that FUS undergoes CRL4B-mediated polyubiquitination and proteasomal degradation, supporting the conclusion that FUS serves as a novel substrate for the CRL4B E3 ubiquitin ligase complex in HCC cells.

FUS plays a critical role in the biogenesis of a specific subset of miRNAs, including miR-143-3p, and its depletion reduces miR-143-3p expression levels [49]. Indeed, FUS knockdown *via* siRNA significantly reduced miR-143-3p levels in Huh7 and LM3 cells (Fig. 4F). Given that CUL4B knockdown induces FUS accumulation, the potential involvement of FUS in mediating the increase in miR-143-3p expression caused by CUL4B knockdown was further investigated. Silencing FUS in CUL4B-knockdown cells, which exhibited elevated FUS levels, effectively blocked miR-143-3p upregulation (Fig. 4G), indicating that FUS mediates miR-143-3p regulation by CUL4B. To further investigate its role in cell growth regulation by CUL4B, *FUS* was silenced in CUL4B-knockdown Huh7 cells using siRNA transfection. FUS knockdown promoted cell growth, consistent with the observation that miR-143-3p inhibition enhanced tumor cell proliferation (Fig. 4H). The consistent observation that FUS knockdown reduces miR-143-3p levels and promotes cell growth, supports a model wherein the FUS/miR-143-3p axis likely functions as a tumor suppressor cascade in the context of HCC progression. Moreover, CCK8 assays demonstrated that the tumor-suppressive effects of CUL4B knockdown were significantly diminished by FUS inhibition (Fig. 4I). Overall, these data indicate that the pro-survival function of CUL4B relies on negative regulation of the FUS/miR-143-3p axis.

### FUS is a ubiquitin substrate of CRL4B^DTL^ E3 ligase

**Fig. 5:**
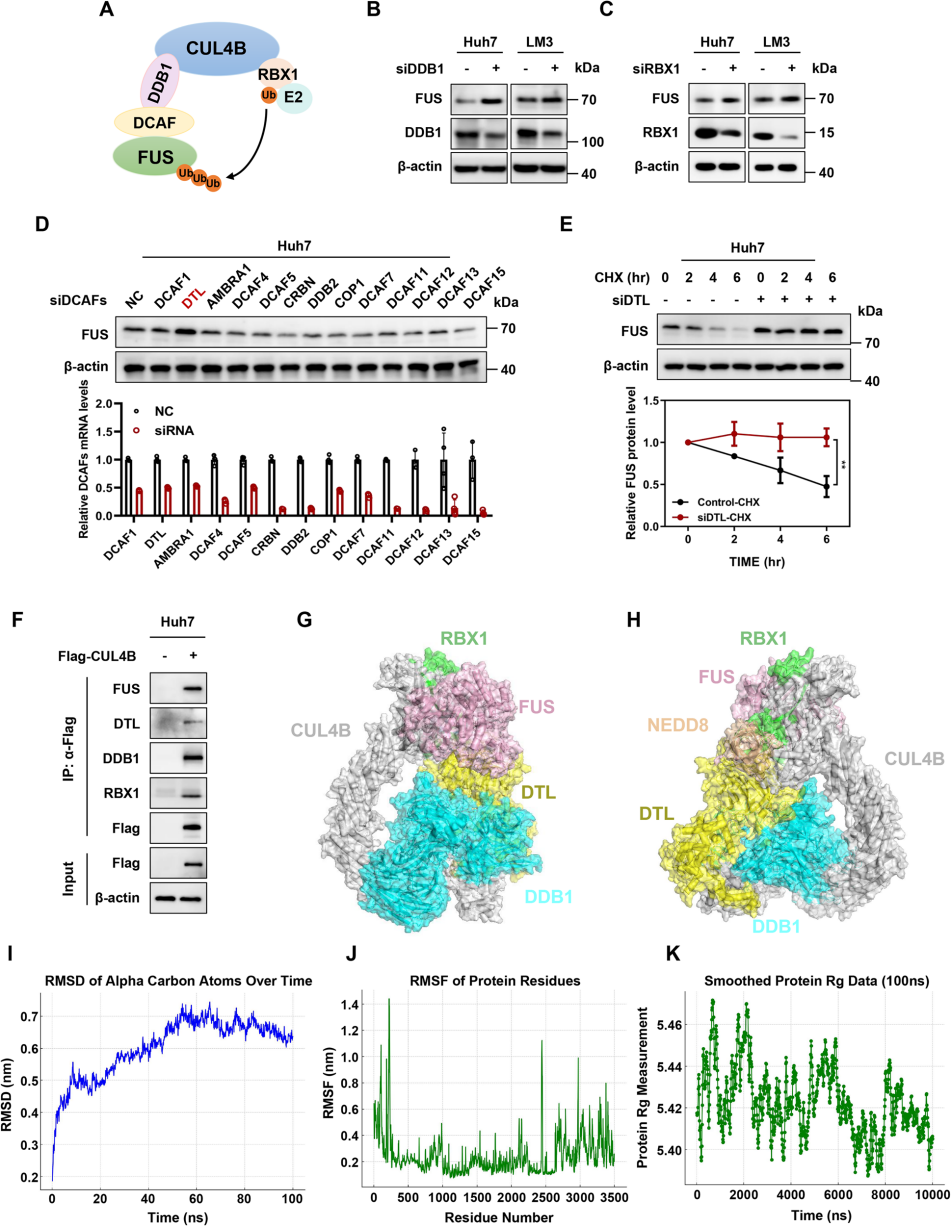
FUS is a ubiquitin substrate of CRL4B^DTL^ E3 ligase. **A** Structural model of CRL4B E3 ligase. **B** FUS protein level in DDB1-knockdown cells. Huh7 and LM3 cells were transfected with control or DDB1 siRNA for 72 h and then harvested for western blotting analysis. **C** FUS protein level in RBX1-knockdown cells. Huh7 and LM3 cells were transfected with control or RBX1 siRNA for 72 h and then harvested for western blotting analysis. **D** FUS protein level in DCAFs-knockdown Huh7 cells. Huh7 cells were transfected with control or DCAF siRNAs for 72 h and then harvested for western blotting analysis. **E** Half-life of FUS in DTL-knockdown cells. Huh7 cells were transfected with control or DTL siRNA for 72 h and then treated with 50 μg/mL CHX at the indicated time points before being subjected to western blotting analysis. FUS protein expression was quantified using ImageQuant TL software, with levels normalized to that of β-actin for comparison (mean ± SD, *n* = 3). **F** The interaction among CUL4B, DTL, DDB1, RBX1, and FUS. Vectors encoding Flag-tagged CUL4B were transfected into Huh7 cells. Cells were then subjected to immunoprecipitation with anti-Flag M2 affinity resin and western blotting analysis. **G**, **H** Molecular docking model of CRL4B^DTL^ and FUS based on AlphaFold3 protein prediction. **I** Root Mean Square Deviation (RMSD) of CRL4B^DTL^-FUS complex structure during 100 ns MD simulation. **J** Root Mean Square Fluctuation (RMSF) of CRL4B^DTL^-FUS complex residues during 100 ns MD simulation. **K** Radius of Gyration (RG) of CRL4B^DTL^-FUS complex structures during 100 ns MD simulation. Two-tailed, unpaired *t*-test was used for (**E**). Data are presented as the mean ± standard deviation derived from a minimum of three independent experiments. Statistical significance was assessed using the appropriate testing methods, with **p < 0.01 denoting levels of significance.

CUL4B functions as a scaffold protein, assembling various CRL4B complexes (Fig. 5A) [24]. Depletion of either DDB1 or RBX1 individually led to FUS accumulation in liver cancer cells (Fig. 5B, C). To identify specific DCAFs targeting FUS, we silenced 13 well-characterized receptor proteins using siRNAs. Notably, DTL knockdown significantly increased FUS levels and prolonged its half-life (Fig. 5D, E). To directly elucidate the interaction between the CUL4B-DDB1-RBX1-DTL complex (CRL4B^DTL^) and FUS, we conducted co-immunoprecipitation assays utilizing an anti-Flag-CUL4B antibody for exogenous pulldown. This methodology clearly demonstrated the interactions among CUL4B, DTL, DDB1, RBX1, and FUS (Fig. 5F).

To comprehensively elucidate the regulatory mechanism between CRL4B^DTL^ and FUS from a structural perspective, we conducted extensive molecular docking and molecular dynamics (MD) simulations using the advanced AlphaFold3-predicted protein structure. The structural diagrams of molecular docking from two different perspectives were presented in Fig. 5G, H. Through rigorous computational analysis of the AlphaFold3-predicted protein structure model, we obtained several key structural insights. First, the Root Mean Square Deviation (RMSD) analysis of the CRL4B^DTL^-FUS complex demonstrated that the system achieved equilibrium and maintained a stable conformation throughout the simulation period (Fig. 5I), which indicating the reliability of our structural model for further analysis. Second, Root Mean Square Fluctuation (RMSF) analysis revealed that the core regions of FUS, particularly those involved in the interaction interface with CRL4B^DTL^, exhibited remarkably low fluctuation values (Fig. 5J). This observation strongly suggests that these regions maintain exceptional structural stability and are significantly constrained by the complex formation, highlighting the specificity of the interaction. Third, the Radius of gyration (Rg) analysis showed that FUS maintained a consistent and compact structure throughout the simulation (Fig. 5K). The stable Rg values indicate that FUS does not undergo large-scale unfolding or structural collapse when bound to the CRL4B^DTL^ complex, maintaining its native conformational compactness. These comprehensive structural analyses collectively demonstrate that the CRL4B^DTL^ complex can stably and specifically interact with FUS. The detailed structural characterization provides crucial insights into the molecular basis of their interaction, explaining how CRL4B^DTL^ exerts its ubiquitin ligase activity to promote FUS ubiquitination and subsequent degradation.

### CUL4B/FUS/miR-143-3p axis regulates KRAS signaling

**Fig. 6:**
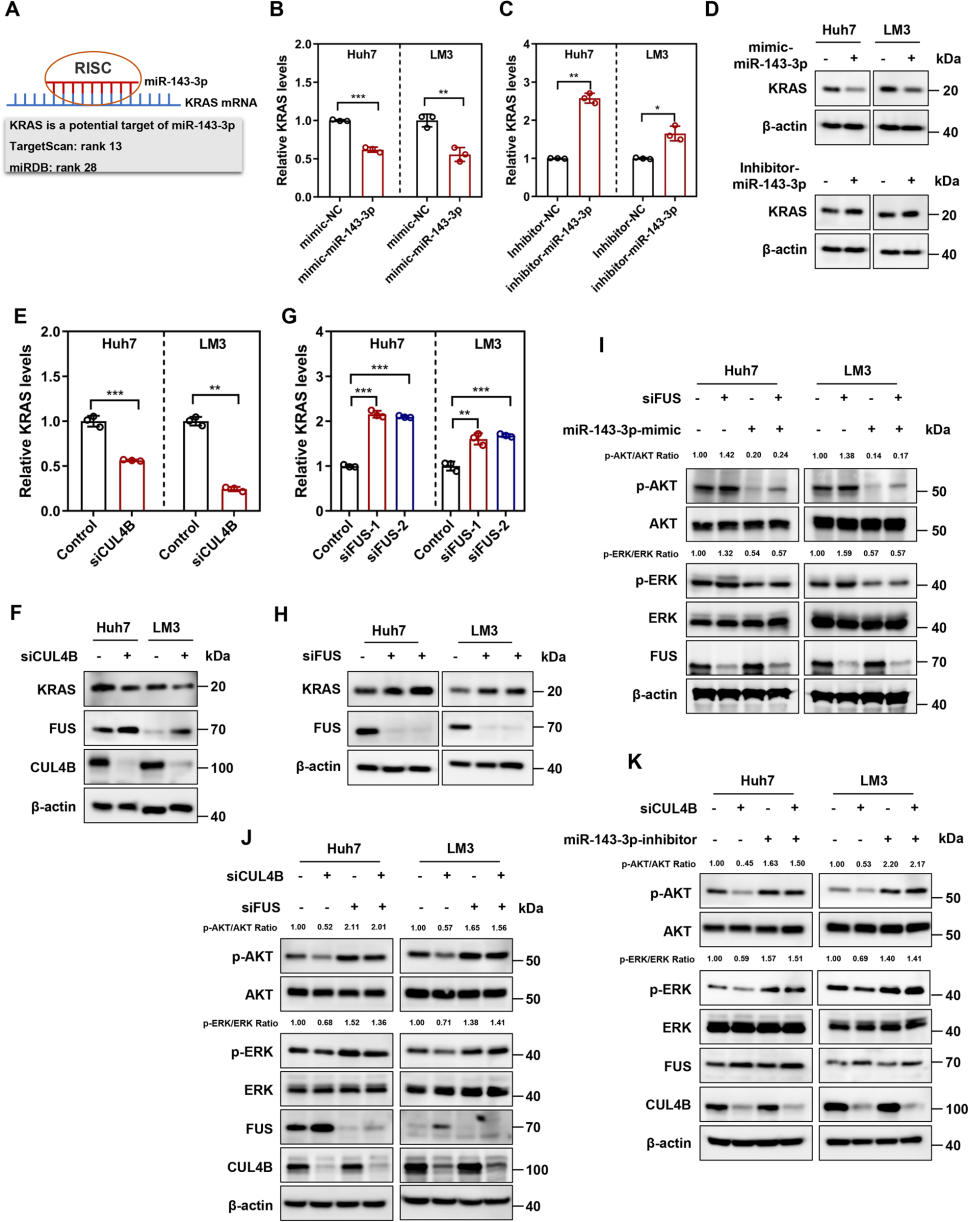
CUL4B/FUS/miR-143-3p axis regulates KRAS signaling. **A** TargetScan and miRDB databases predict KRAS as a potential target of miR-143-3p. **B**, **C** KRAS mRNA level determined using qRT-PCR after transfecting Huh7 and LM3 cells with mimic-miR-143-3p (**B**) or inhibitor-miR-143-3p (**C**) for 72 h (mean ± SD, *n* = 3). **D** KRAS protein level determined using western blotting after transfecting Huh7 and LM3 cells with mimic-miR-143-3p or inhibitor-miR-143-3p for 72 h. (**E**, **F**) KRAS mRNA and protein levels determined using qRT-PCR and western blotting, respectively, after CUL4B knockdown (mean ± SD, *n* = 3). **G**, **H** KRAS mRNA and protein levels determined using qRT-PCR and western blotting, respectively, after FUS knockdown (mean ± SD, *n* = 3). **I** Levels of phosphorylated AKT and phosphorylated ERK determined using western blotting after transfecting FUS-knockdown cells with miR-143-3p mimic for 72 h. **J**, **K** Levels of phosphorylated AKT and phosphorylated ERK determined using western blotting after transfecting CUL4B-knockdown cells with FUS siRNA or miR-143-3p inhibitor for 72 h. Two-tailed, unpaired *t*-test was used for (**B**−**E**). One-way ANOVA/LSD test was used for (**G**). Data are presented as the mean ± standard deviation derived from a minimum of three independent experiments. Statistical significance was assessed using the appropriate testing methods, with *p < 0.05, **p < 0.01, and ***p < 0.001 denoting levels of significance.

To elucidate how the CUL4B/FUS/miR-143-3p axis influences HCC growth and oxaliplatin sensitivity, potential miR-143-3p targets were investigated. Bioinformatic analysis using TargetScan and miRDB predicted miR-143-3p binding sites within the KRAS 3ʹ-UTR, identifying KRAS as a putative target (Fig. 6A). Supporting KRAS’s oncogenic role in HCC, its expression was significantly upregulated in TCGA HCC tissues compared to normal tissues, and higher KRAS levels correlated with poorer patient survival (Supplemental Figs. 5A, B). Bioinformatics also revealed a strong positive correlation between CUL4B and KRAS mRNA levels (Supplemental Fig. 5C). Functional validation demonstrated that siRNA-mediated KRAS knockdown alone significantly inhibited Huh7 cell proliferation and enhanced oxaliplatin sensitivity (Supplemental Figs. 5D, E). Subsequent experiments confirmed that miR-143-3p directly regulates KRAS, as its overexpression reduced, while its inhibition increased, KRAS expression at both mRNA and protein levels (Fig. 6B–D). Given that CUL4B suppresses miR-143-3p by degrading FUS, we assessed the impact of this axis on KRAS. Consistent with the model, CUL4B knockdown decreased KRAS expression (Fig. 6E, F), whereas FUS knockdown conversely increased it (Fig. 6G, H), demonstrating that the CUL4B/FUS axis inversely controls KRAS levels via miR-143-3p.

While the interplay between KRAS signaling, ID1/c-MYC, and chemoresistance is acknowledged [50–52], we specifically assessed whether CUL4B depletion affects ID1 and c-MYC expression. However, CUL4B knockdown did not alter ID1 or c-MYC protein levels (Supplemental Fig. 6). Although ID1 and c-MYC are well-established mediators of oxaliplatin resistance, these findings suggest that CUL4B-mediated regulation of KRAS signaling and its influence on HCC oxaliplatin sensitivity may function independently of the ID1/c-MYC axis.

Further analysis of downstream KRAS effectors showed that FUS knockdown increased ERK and AKT phosphorylation, an effect reversed by miR-143-3p overexpression (Fig. 6I). Conversely, CUL4B knockdown decreased ERK and AKT phosphorylation, but this suppression was reversed by either FUS knockdown or miR-143-3p inhibition (Fig. 6J, K). Collectively, these findings demonstrate that the CUL4B/FUS/miR-143-3p axis promotes HCC growth and oxaliplatin resistance, at least partially, by activating the KRAS signaling pathway.

## Discussion

**Fig. 7:**
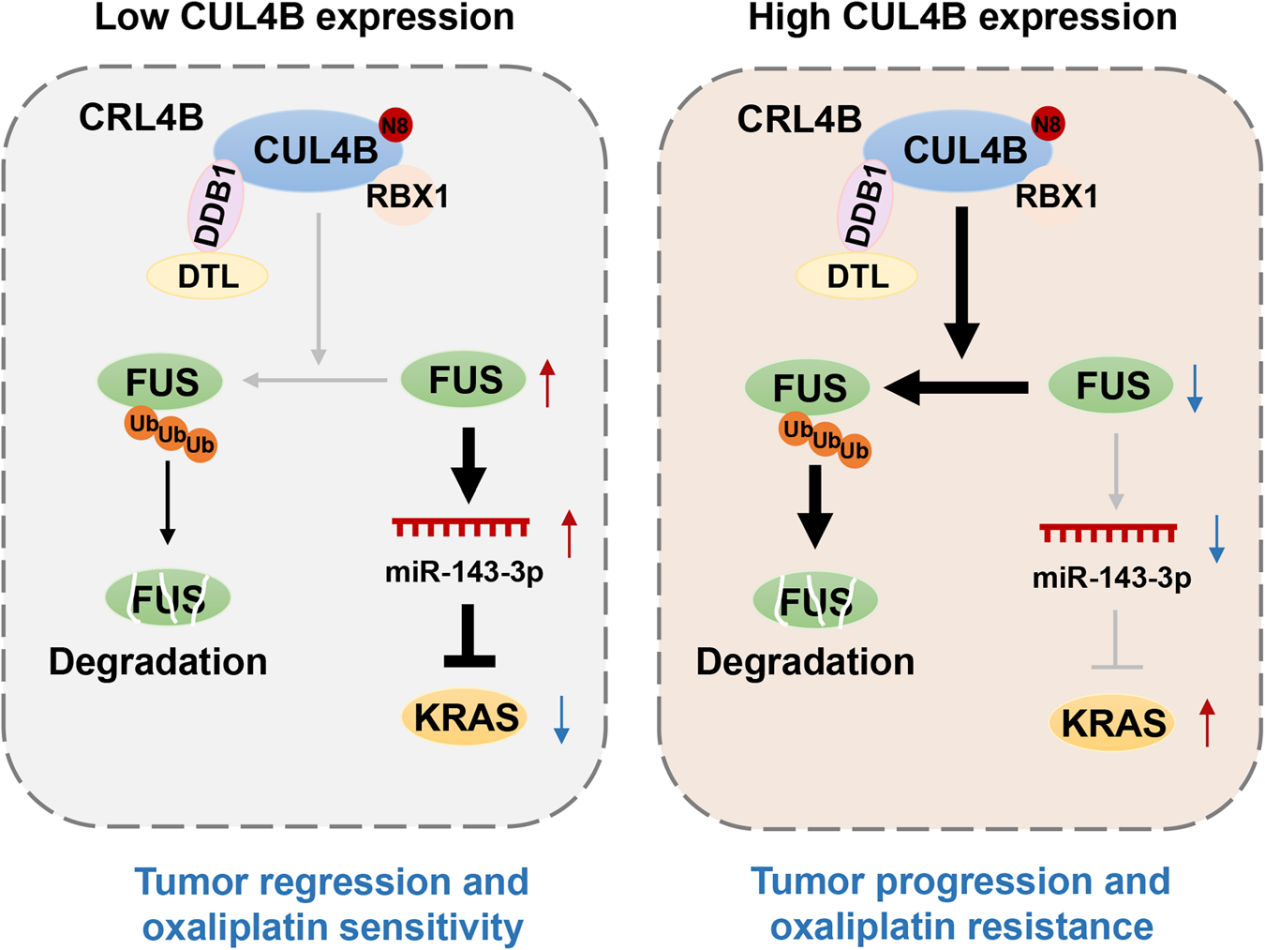
Proposed working model for the role of the CUL4B/FUS/miR-143-3P/KRAS axis in HCC growth and oxaliplatin resistance. Aberrantly activated CUL4B promotes FUS polyubiquitination and degradation, leading to downregulation of miR-143-3p and activation of the KRAS signaling pathway, thus promoting HCC progression and oxaliplatin resistance.

HCC remains a highly lethal malignancy with limited therapeutic options, particularly for patients with advanced disease. Oxaliplatin-based regimens are widely used in the treatment of advanced HCC, yet their efficacy is frequently compromised by intrinsic or acquired resistance [5]. In this study, we identified the CUL4B/FUS/miR-143-3p/KRAS axis as a key mechanism underlying HCC progression and oxaliplatin resistance (Fig. 7). Our data suggest that targeting CUL4B may represent a promising strategy to suppress tumor growth and restore oxaliplatin sensitivity in HCC.

CUL4B has been previously reported to function as an oncogene in several solid tumors. For instance, in breast cancer, CUL4B facilitates hypoxia-induced tumor progression through transcriptional repression [26], while in prostate cancer, it forms a positive feedback loop with SOX4 to drive malignancy [31]. In bladder cancer, CUL4B promotes metastasis via the miR-372/373-PIK3CA-AKT cascade [32]. Despite these advances, the role of CUL4B in HCC, particularly its involvement in chemotherapy resistance, has remained unclear. Our study demonstrates that CUL4B promotes oxaliplatin resistance in HCC through a previously uncharacterized mechanism involving FUS degradation and subsequent miR-143-3p downregulation.

Our initial miRNA profiling revealed multiple candidate miRNAs regulated by neddylation. Among these, miR-143-3p stood out due to its well-documented tumor-suppressive functions across various cancers [41–44]. Although other miRNAs such as miR-365a-5p also exhibited significant changes, our functional studies consistently supported miR-143-3p as a critical downstream effector of CUL4B in HCC. Knockdown of CUL4B robustly upregulated miR-143-3p, and inhibition of miR-143-3p effectively reversed the anti-tumor effects of CUL4B silencing. Importantly, CUL4B-mediated oxaliplatin resistance was dependent on miR-143-3p suppression, as restoring miR-143-3p expression resensitized cells to treatment. While we cannot exclude the possibility that CUL4B-FUS axis may also regulate other miRNAs (e.g., miR-365a-5p), our functional data strongly support miR-143-3p as a central player in CUL4B-driven oncogenesis.

The role of miR-143-3p in drug resistance appears to be context-dependent. In prostate cancer, miR-143-3p enhances sensitivity to abiraterone acetate by suppressing JNK signaling [53], while in triple-negative breast cancer, it reverses gemcitabine resistance by inhibiting glycolysis [54]. Conversely, in melanoma, miR-143-3p has been reported to reduce the efficacy of targeted therapies [55]. Our findings indicate that in HCC, miR-143-3p acts as a chemosensitizer by directly targeting KRAS. Although KRAS mutations are rare in HCC, wild-type KRAS can be activated by upstream signals and contribute to HCC pathogenesis [56, 57]. Previous studies have shown that miR-143-3p suppresses both wild-type and mutant KRAS in colorectal cancer [45]. Similarly, our results demonstrate that CUL4B knockdown reduces KRAS expression in a miR-143-3p-dependent manner, establishing a direct link between CUL4B and KRAS signaling via miR-143-3p.

Mechanistically, we identified FUS as a novel substrate of the CRL4B^DTL^ complex. While CRL4B is best known for its role in epigenetic silencing through H2AK119 mono-ubiquitination and recruitment of polycomb repressive complex 2 (PRC2) [25, 58], our study reveals an alternative proteolytic mechanism whereby CUL4B promotes the polyubiquitination and degradation of FUS. FUS has context-dependent roles in cancer: it acts as an oncoprotein in sarcomas through fusion events such as FUS-CHOP [59, 60], but exhibits tumor-suppressive properties in liver, prostate, and breast cancers [61–63]. In HCC, FUS loss has been linked to enhanced invasion and metastasis via RhoA/Rac1 activation [61]. Moreover, FUS is known to facilitate the processing of pri-miR-143-3p by recruiting the Drosha complex [49]. Our data align with these findings, supporting a tumor-suppressive role for FUS in HCC through its positive regulation of miR-143-3p.

This study has several limitations warranting consideration. Primarily, experimental validation through protein crystallization and structural analysis of the CRL4B^DTL^-FUS complex using techniques like X-ray crystallography or cryo-EM would significantly strengthen and refine our computational predictions. Secondly, while FUS exhibits pro-oncogenic effects here, its role in positively regulating miR-143-3p expression across other cancer types remains unclear. Thirdly, given FUS’s extensive involvement in diverse RNA processes beyond miRNA maturation, such as mRNA splicing and circular RNA formation, further investigation is essential to determine if CUL4B influences tumorigenesis through these additional FUS-mediated pathways. Finally, exploring the potential link between CUL4B expression and oxaliplatin-based hepatic artery infusion chemotherapy (HAIC) efficacy in HCC patients holds considerable clinical value; however, direct validation within this study faces substantial hurdles, including the challenge of securing a large, well-annotated patient cohort with detailed HAIC records and paired biospecimens, alongside ethical constraints.

In conclusion, this study highlights the oncogenic role of CUL4B in liver cancer and its potential as a target for enhancing oxaliplatin sensitivity in HCC cells. This effect is mediated by promoting FUS polyubiquitination and degradation, reducing miR-143-3p levels, and subsequently activating the KRAS signaling pathway (Fig. 7). These findings validate CUL4B as a promising therapeutic target for liver cancer treatment and a potential sensitizing target for oxaliplatin.

## Materials and Methods

### Cell culture

The human liver cancer cell lines, Huh7 and LM3, were sourced from the National Collection of Authenticated Cell Cultures, and the human renal epithelial cell line, HEK293T, was obtained from the American Type Culture Collection. All three cell lines were cultured in Dulbecco’s Modified Eagle’s medium (DMEM) (BasalMedia, L110KJ) supplemented with 10% fetal bovine serum (FBS) (ExCell Bio, FSP500) and 1% penicillin–streptomycin solution (BasalMedia, S110JV). All cell lines were subjected to at least five to six passages before experimental use, verified to be mycoplasma-free, and maintained in a humidified incubator at 37 °C with 95% air and 5% carbon dioxide.

### Antibodies and plasmids

Commercially sourced antibodies were diluted before use as follows: ADAR1 (Abcam, ab307585), 1:1000; AKT (Cell Signaling Technology, 9272), 1:1000; β-actin (Proteintech, 66009-1-Ig), 1:10000; c-MYC (Proteintech, 10828-1-AP), 1:1000; c-PARP (Cell Signaling Technology, 5625), 1:1000; Cullin1 (Abcam, 75817), 1:1000; Cullin2 (Abcam, 166917), 1:1000; Cullin3 (Cell Signaling Technology, 2759), 1:1000; Cullin4A (Cell Signaling Technology, 2699), 1:1000; Cullin4B (Proteintech, 12916-1-AP), 1:1000; Cullin5 (Abcam, 184177), 1:1000; Cyclin B (Cell Signaling Technology, 4138), 1:1000; DDB1 (Abcam, ab109027), 1:1000; Dicer (Abcam, ab315232), 1:1000; DGCR8 (Abcam, ab191875), 1:1000; Drosha (Abcam, ab183732), 1:1000; DTL (Abcam, ab306556), 1:1000; ID1 (Cell Signaling Technology, 23369), 1:1000; RBX1 (Abcam, ab133565), 1:1000; ERK (Cell Signaling Technology, 4695), 1:1000; FUS (Abcam, ab124923; Cell Signaling Technology, 67840), 1:1000; KRAS (Cell Signaling Technology, 33197), 1:1000; NOXA (Cell Signaling Technology, 14766), 1:1000; p-AKT (Cell Signaling Technology, 4060), 1:1000; p-ERK (Cell Signaling Technology, 4370), 1:1000; and Ub (Cell Signaling Technology, 20326), 1:1000. Flag-FUS (HG16569-CF) and Flag-CUL4B (HG14423-CF) plasmids were obtained from SinoBiological. cDNA-encoding full-length human Ub was cloned into pCMV3-HA-tagged vectors.

### Generation of stable CUL4B-knockdown cell lines using CRISPR/Cas9

CUL4B-knockdown cells were generated using CRISPR-Cas9 technology as described previously [64, 65]. Single guide RNAs (sgRNAs) were designed using an online tool at crispr.mit.edu to target the human *CUL4B* gene. Complementary sgRNA oligos were synthesized by Sangon Biotech (Shanghai, China), annealed to form double-stranded DNA, and cloned into a BsmBI-digested lenti-Guide-CRISPR-v2-puro vector. The cloned fragments were sequenced to verify their accuracy. The vector containing either nontarget control (NC) or CUL4B-targeting gRNA (4.0 μg) was co-transfected with psPAX2 (3.0 μg) and pMD2.G plasmids (1.0 μg) into HEK293T cells using Lipofectamine 3000 (Invitrogen, USA) according to the manufacturer’s instructions. After 48 h from transfection, viral supernatants were harvested, filtered, and mixed with 10 μg/mL polybrene (Beyotime, C0351) to enhance the infection efficiency. The multiplicity of infection (MOI) was optimized to 15 based on preliminary titration experiments. Huh7 and LM3 cells, seeded at 40% confluency in six-well plates, were infected with viral supernatants for 48 h. Infected cells were selected with 10 μg/mL puromycin (Beyotime, ST551) for 5 days, with complete death of non-infected control cells confirming effective selection, and knockdown efficiency was validated *via* western blotting. The sequence of the NC oligo was 5ʹ-ACGGAGGCTAAGCGTCGCAA-3ʹ, whereas those of the CUL4B-targeting sgRNA oligos (CUL4B#1 and CUL4B#2) were 5ʹ-AAAACTACACAGATGAAACC-3ʹ and 5ʹ-CAATTTAGAAGAACTCTACC-3ʹ, respectively.

### Cell proliferation and IC_50_ analysis

For proliferation assays, cells were seeded in 96-well plates at a density of 3000 cells/well and monitored over four consecutive days, with or without oxaliplatin treatment (2 μM), using CCK8 (Dojindo, CK04), according to the manufacturer’s instructions.

For IC_50_ analysis, cells were seeded in 96-well plates at a density of 3000 cells/well and treated with varying concentrations of oxaliplatin (0, 1.25, 2.5, 5, 10, 20, 40, 80, and 160 μM) for 48 h. Cell viability was assessed using an ATPlite luminescence assay kit (PerkinElmer, USA), following the manufacturer’s protocol.

### Protein half-life analysis

Cells were transfected with siCUL4B oligo for 72 h or treated with 1 μM MLN4924 for 24 h. Following these treatments, the cells were exposed to 50 μg/mL CHX (Selleck, S7418) for 0, 2, 4, or 6 h. The time points for CHX treatment were selected based on preliminary kinetic studies showing significant FUS degradation within this timeframe. Additionally, the cells were treated with 50 μg/mL CHX in combination with either 10 μM MG132 (Selleck, S2619) or DMSO for the same time intervals. All experiments were performed with three independent biological replicates, and protein quantification was conducted using ImageQuant TL software with normalization to β-actin to ensure accurate half-life determination.

### Flow cytometric analysis of cell cycle and apoptosis

Cells were digested with 0.25% trypsin (without EDTA), and digestion was halted using DMEM supplemented with 10% fetal bovine serum. The cells were then collected, centrifuged at 1200 rpm for 5 min, and washed two times with cold phosphate-buffered saline (PBS). For cell cycle analysis, 5 × 10^5^ cells were fixed in 1 mL of ice-cold 70% ethanol at -20 °C overnight. After centrifugation at 1200 rpm for 5 min and washing two times with cold PBS, the cells were re-suspended in 200 μL cold PBS containing 50 μg/mL propidium iodide (PI; Beyotime, ST1569) and 10 μg/mL RNase A (Beyotime, ST578). Following a 15 min dark incubation at room temperature, cell cycle analysis was conducted using flow cytometry (Beckman Coulter, USA). For apoptosis assays, the Annexin V-FITC Apoptosis Detection Kit (Beyotime, C1062L) was used according to the manufacturer’s instructions. A total of 1 × 10^5^ cells were suspended in 195 μL Annexin V-FITC binding solution containing 5 μL Annexin V-FITC and 10 μL PI. After a 15 min dark incubation at room temperature, the cells were subjected to flow cytometric analysis, with necessary adjustments to voltage and fluorescence compensation. Flow cytometry data were analyzed using the Kaluza Analysis software.

### Animal experiments

BALB/c nude mice were housed in an animal facility under a 12 h light–dark cycle at controlled temperatures of 20–22 °C and 40–70% humidity. All mice were randomly grouped before further experiments. To generate subcutaneous tumor models, 2 × 10^6^ Huh7 cells were injected into 5 week-old male BALB/c nude mice. Tumor tissues were harvested ~20 days later. Once the subcutaneous tumors reached ~5 mm in length, the mice were treated with either oxaliplatin (5 mg/kg every 3 days, administered intraperitoneally) in combination with antagomiR-143-3p or agomiR-143-3p. Tumor dimensions were measured every 3 days using a Vernier caliper, and the size was calculated using the following formula: volume = (length × width^2^)/2. Mice were euthanized by CO_2_ asphyxiation when the tumors in any group reached a length >1.5 cm. The excised tumors were weighed. The oligos were synthesized by GenePharma (Shanghai, China). The 3’ end of antagomiR is modified with cholesterol, the 5’ end with two phosphorothioate linkages, the 3’ end with four phosphorothioate linkages, and the entire chain with methoxy modification. AgomiR is modified on the antisense strand, with cholesterol modification at the 3’ end, two phosphorothioate linkages at the 5’ end, four phosphorothioate linkages at the 3’ end, and methoxy modification throughout the entire chain. The sequences were as follows.

antagomiR-NC: CAGUACUUUUGUGUAGUACAA;

antagomiR-143-3p: GAGCUACAGUGCUUCAUCUCA;

agomiR-NC: UUCUCCGAACGUGUCACGUUU;

agomiR-143-3p: UGAGAUGAAGCACUGUAGCUC.

### Statistical analysis

Data were expressed as mean ± standard deviation (SD) from at least three independent experiments. Statistical analyses and graphical representations were performed using Prism 8 (GraphPad) and SPSS v. 18.0. Differences between groups were assessed using a two-tailed unpaired *t*-test, one-way ANOVA, or two-way ANOVA. *F*-test was first used to compare the variance between the two groups. If the *p*-value of the *F*-test ≥ 0.05, equal variance is assumed when performing the unpaired *t*-test. If the *p*-value of the *F*-test < 0.05, variance is not assumed equally and thus Welch’s correction is applied for unpaired *t*-test. ROC curves were used to determine the optimal cutoff values for CUL4B and miR-143-3p expression during survival analysis. Univariate and multivariate Cox regression and Kaplan–Meier analyses were used to assess the association between CUL4B or miR-143-3p expression and overall survival. Sample sizes were determined based on previous laboratory experience and pre-specified effect sizes deemed biologically significant. No samples or animals were excluded from any analysis except for samples that lacked complete clinical parameters. A blind analysis was not performed. Statistical significance was set at three levels: *p < 0.05, **p < 0.01, and ***p < 0.001.

## Supplementary information

Original Western Blots

Supplemental Figures and Methods

## Data Availability

The data supporting the findings of this study are included in this article. Additional data related to this study are available upon request from the corresponding authors.
