# Supplementary material for: CUL4B promotes hepatocellular carcinoma progression and oxaliplatin resistance by facilitating FUS degradation
Source: Cell Death Dis. 2025 Dec 14;17(1):116. doi: 10.1038/s41419-025-08320-6 (PMC12848007; doi:10.1038/s41419-025-08320-6)

Full unedited blot/gel for Figure 2E

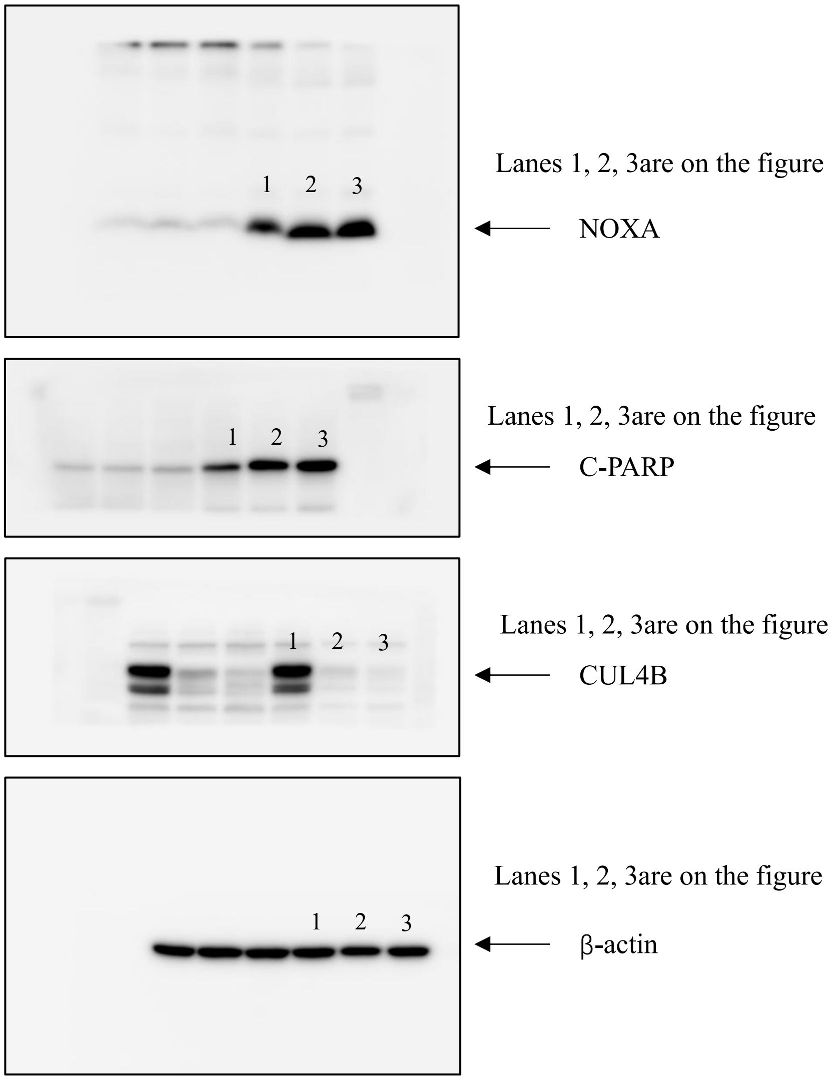

Full unedited blot/gel for Figure 2F

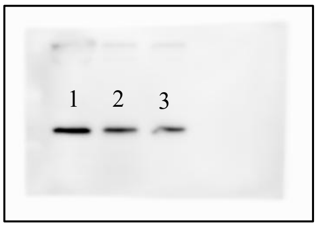

Lanes 1, 2, 3are on the figure

Cyclin B

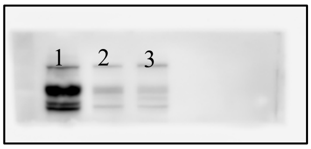

Lanes 1, 2, 3are on the figure

CUL4B

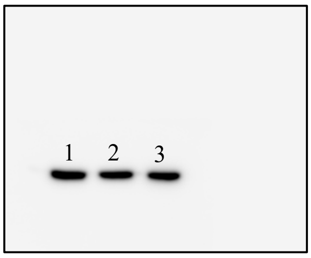

Lanes 1, 2, 3are on the figure

β-actin

Full unedited blot/gel for Figure 4A

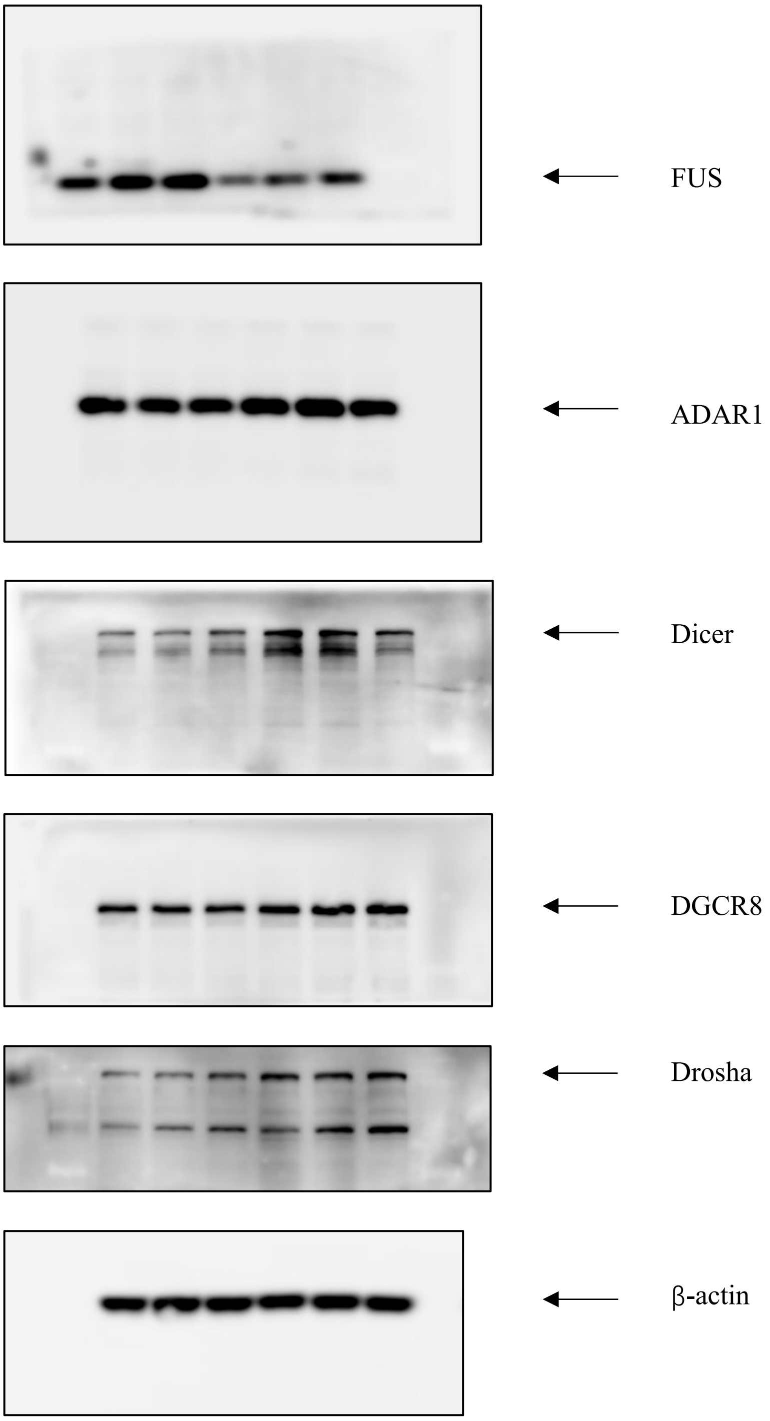

Full unedited blot/gel for Figure 4B

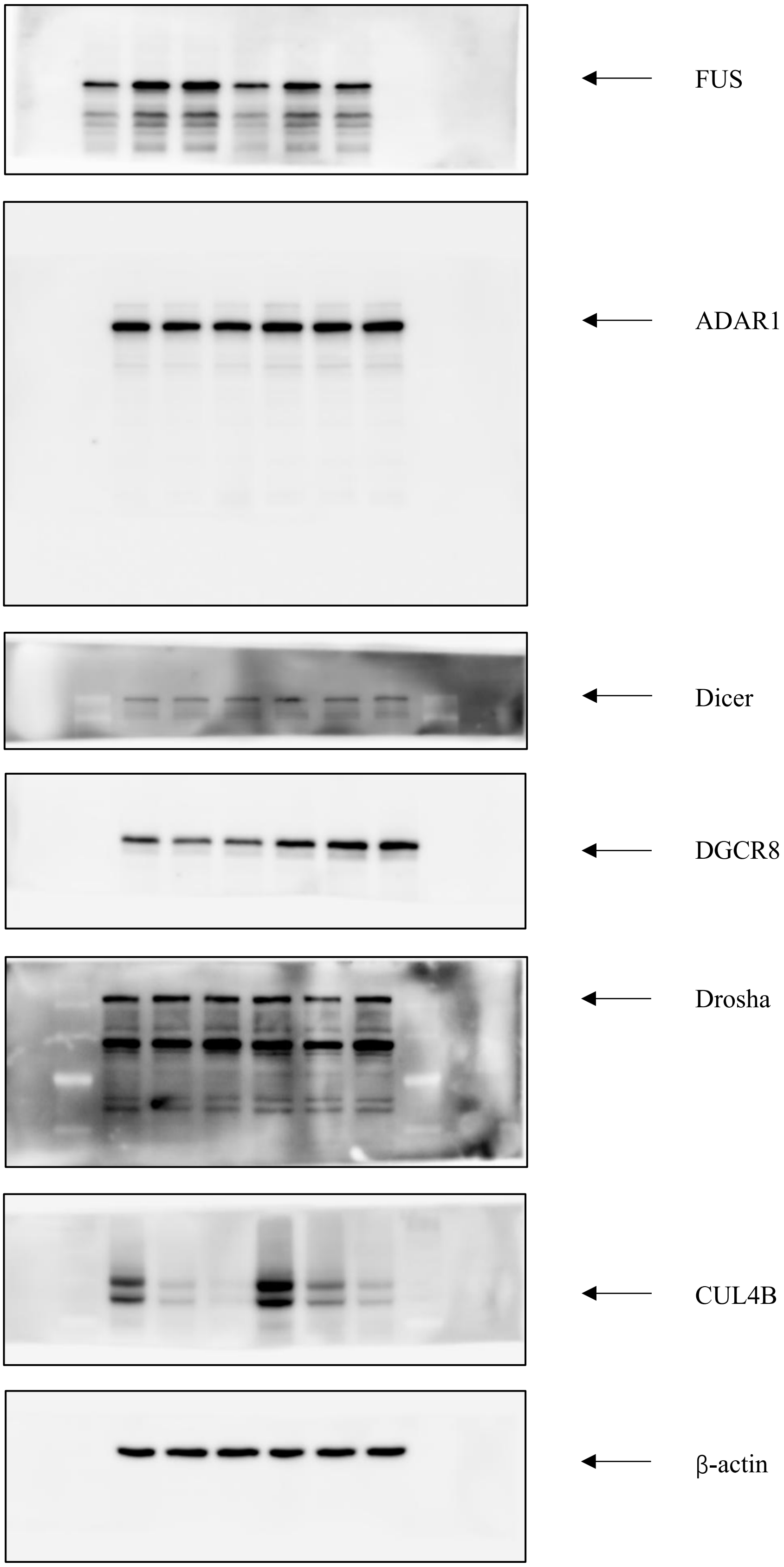

Full unedited blot/gel for Figure 4C

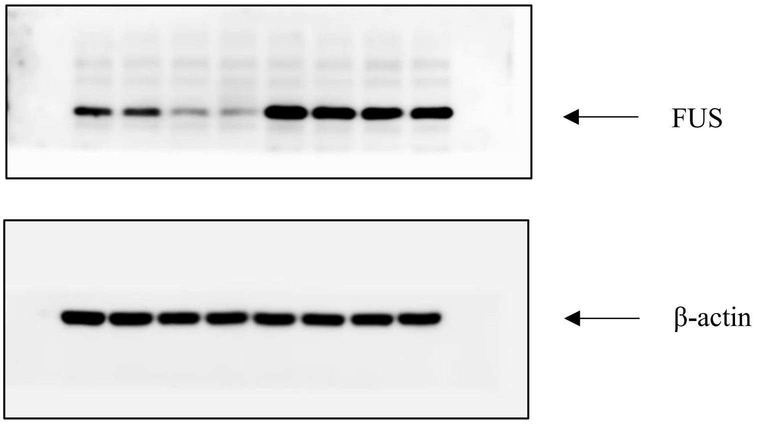

Full unedited blot/gel for Figure 4D

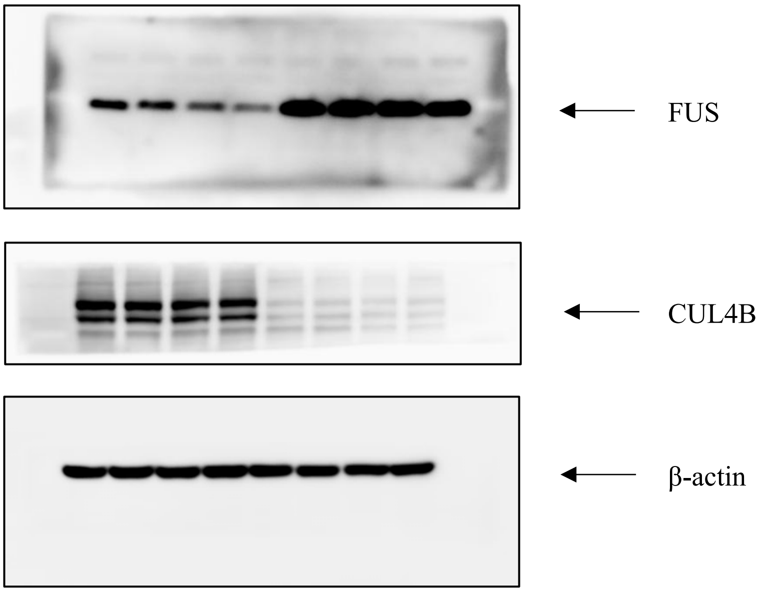

Full unedited blot/gel for Figure 4E

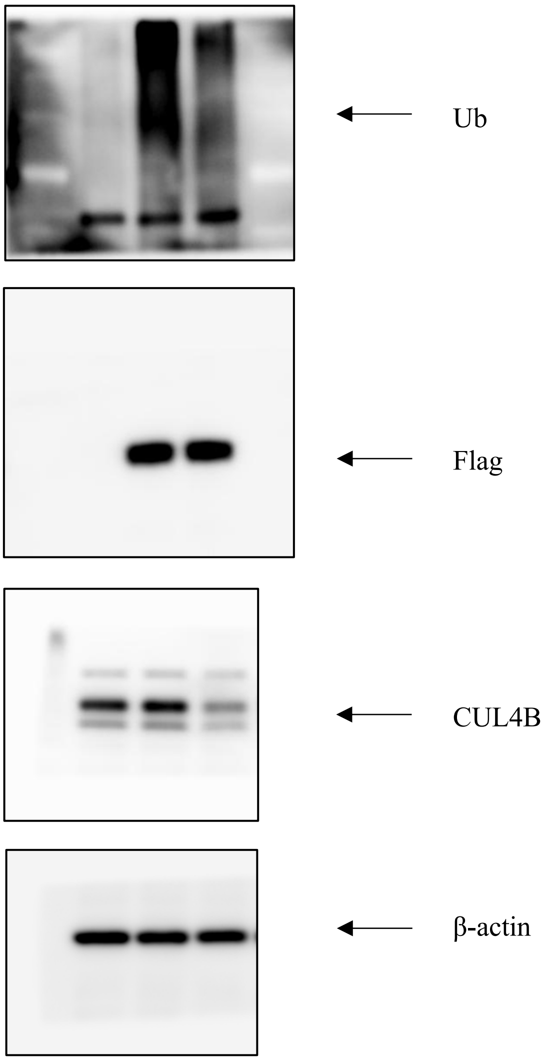

Full unedited blot/gel for Figure 5B

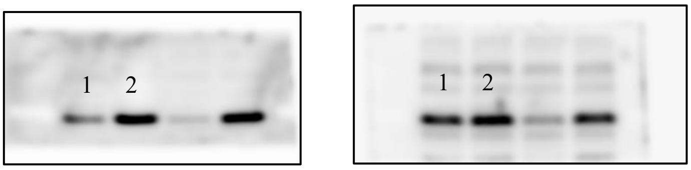

Lanes 1, 2are on the figure

FUS

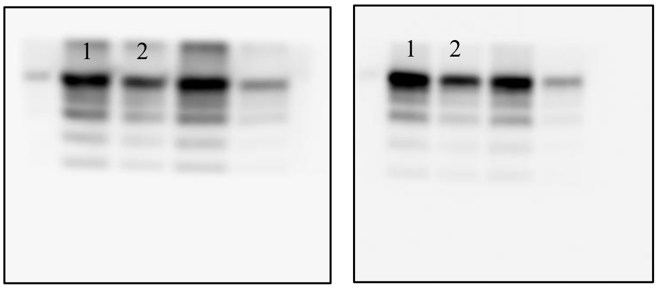

Lanes 1, 2are on the figure

DDB1

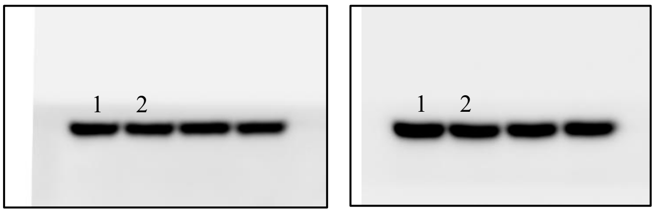

Lanes 1, 2are on the figure

β-actin

Full unedited blot/gel for Figure 5C

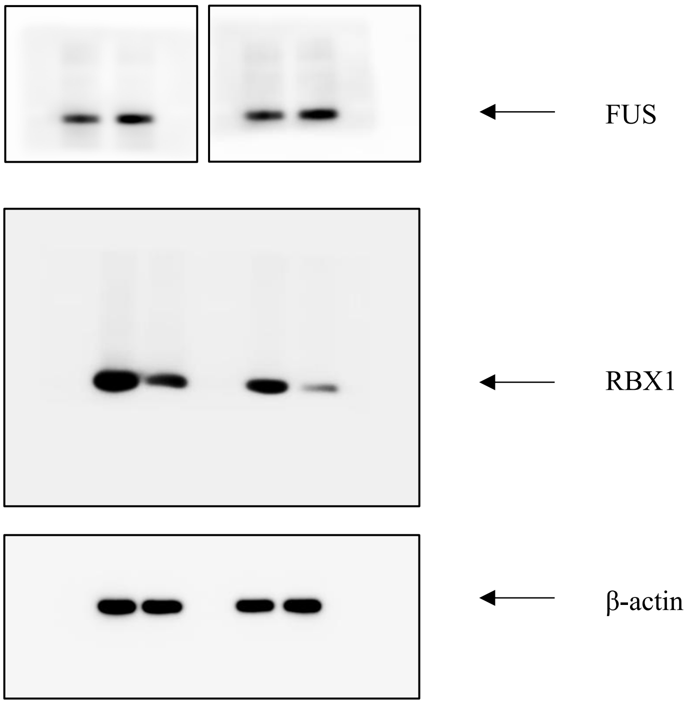

Full unedited blot/gel for Figure 5D

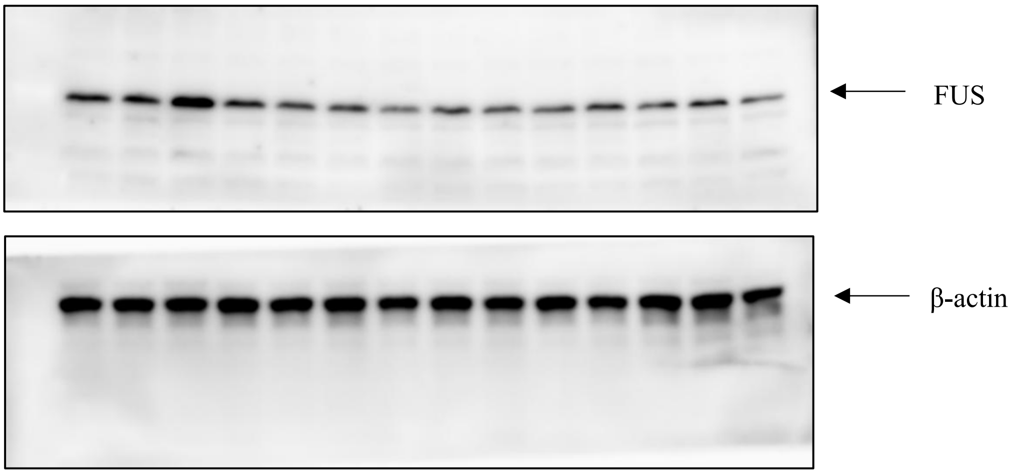

Full unedited blot/gel for Figure 5E

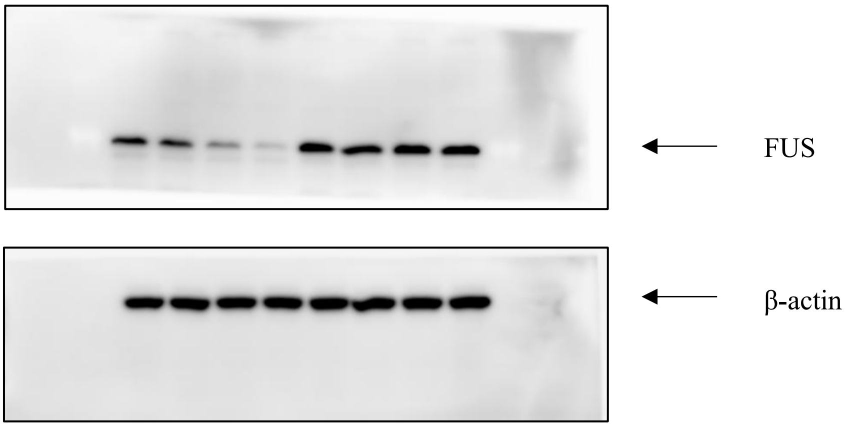

Full unedited blot/gel for Figure 5F

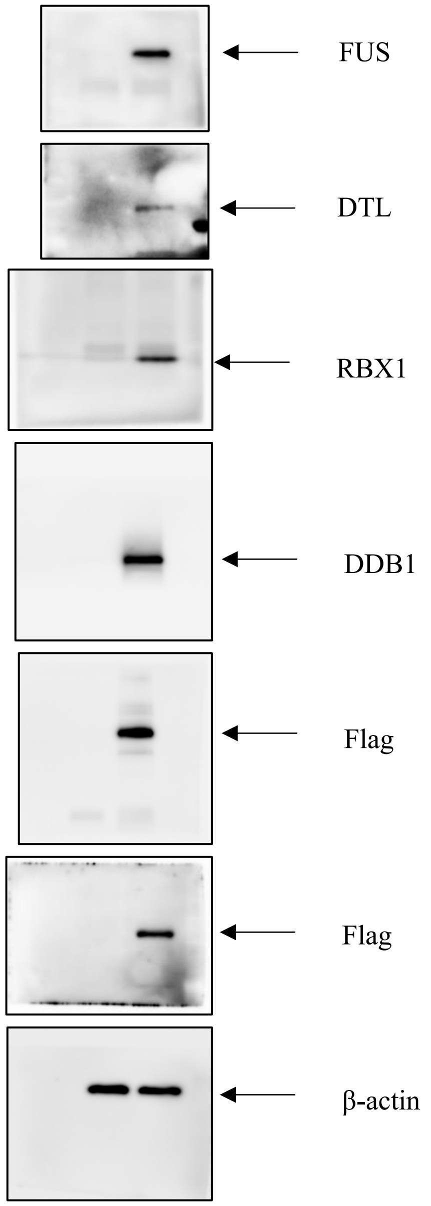

Full unedited blot/gel for Figure 6D

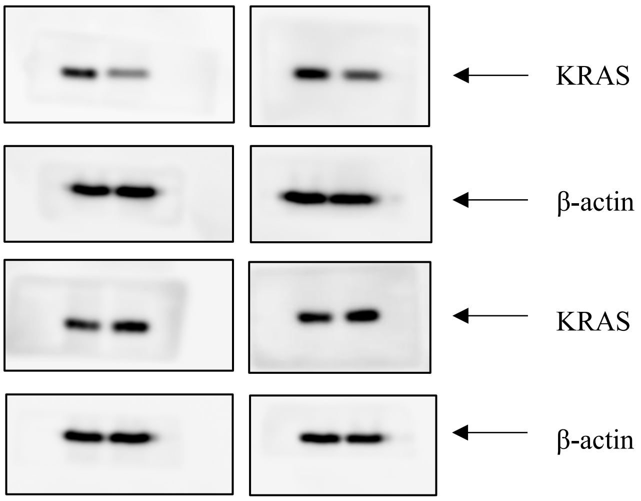

Full unedited blot/gel for Figure 6F

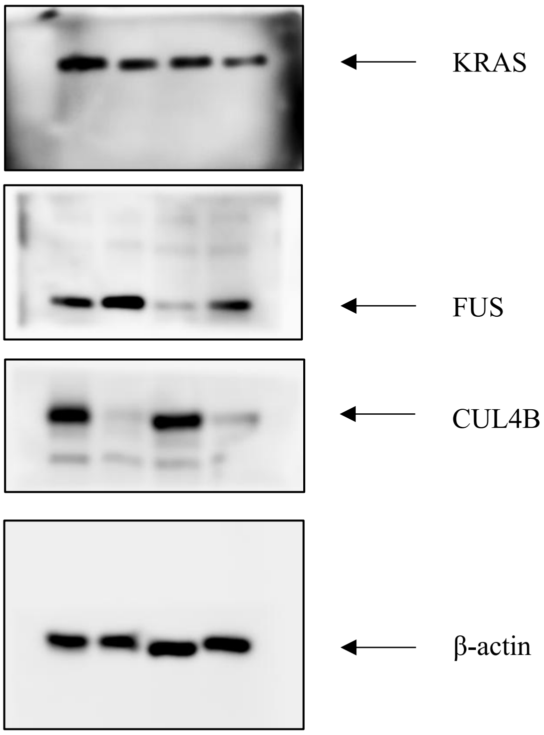

Full unedited blot/gel for Figure 6H

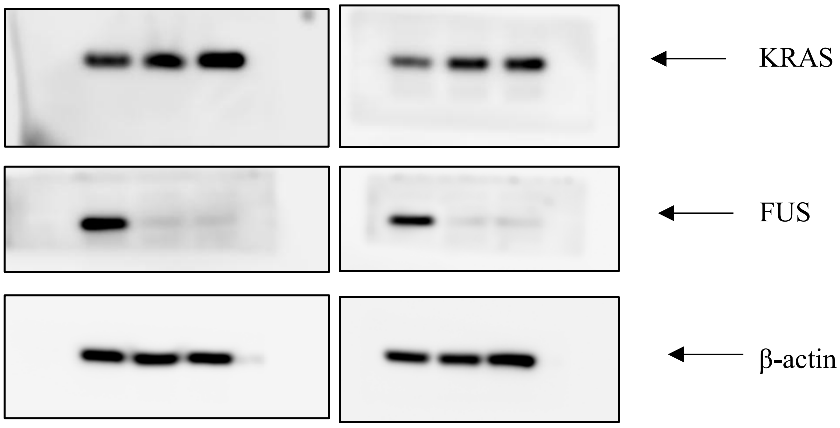

Full unedited blot/gel for Figure 6I

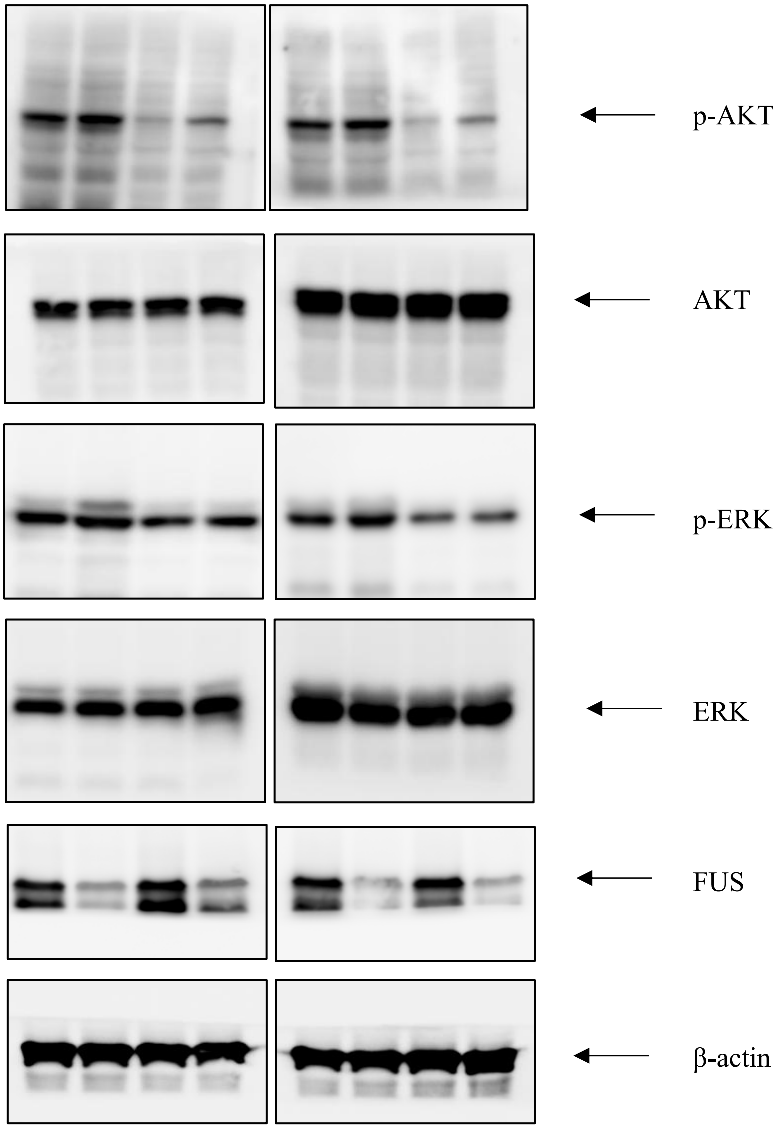

Full unedited blot/gel for Figure 6J

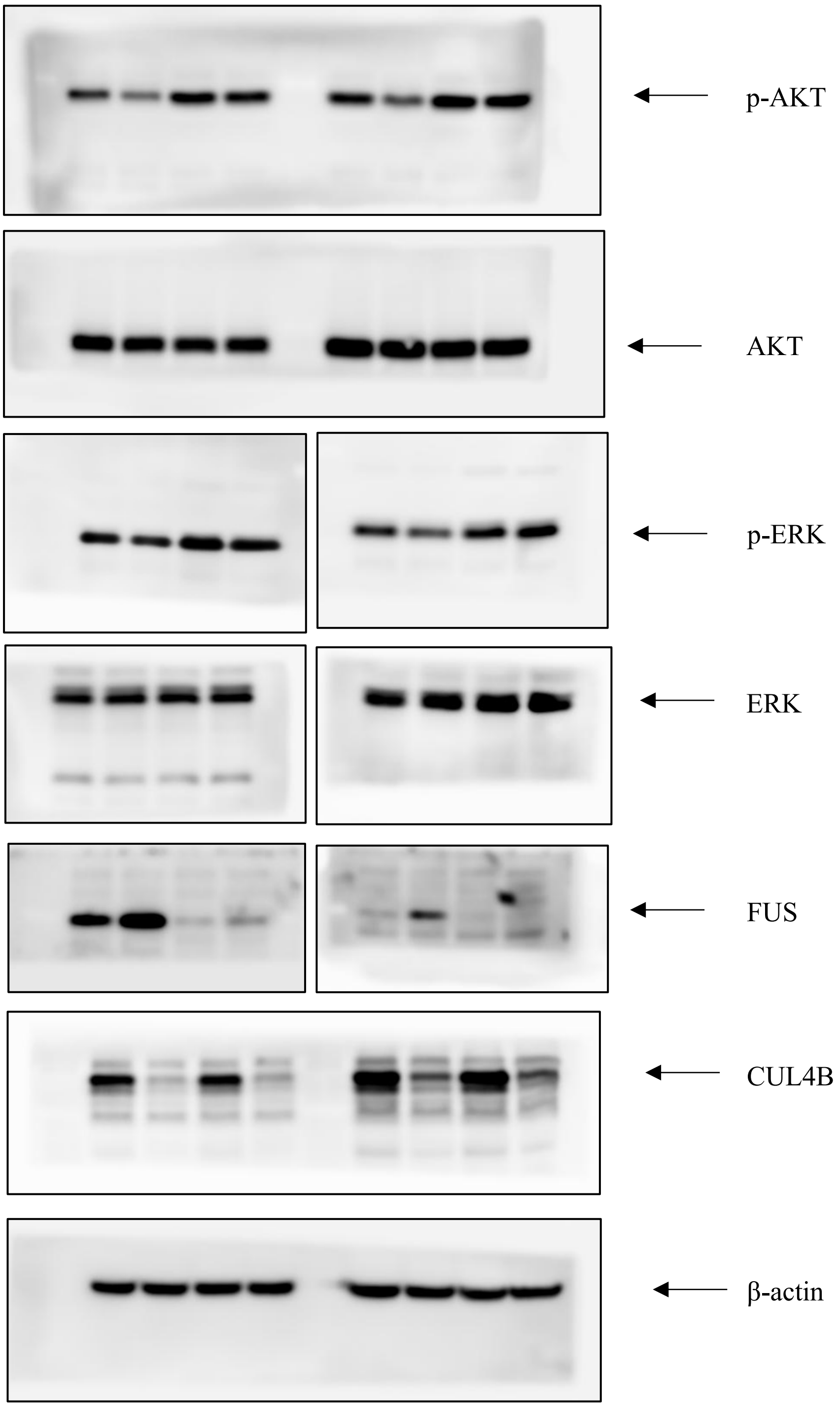

Full unedited blot/gel for Figure 6K

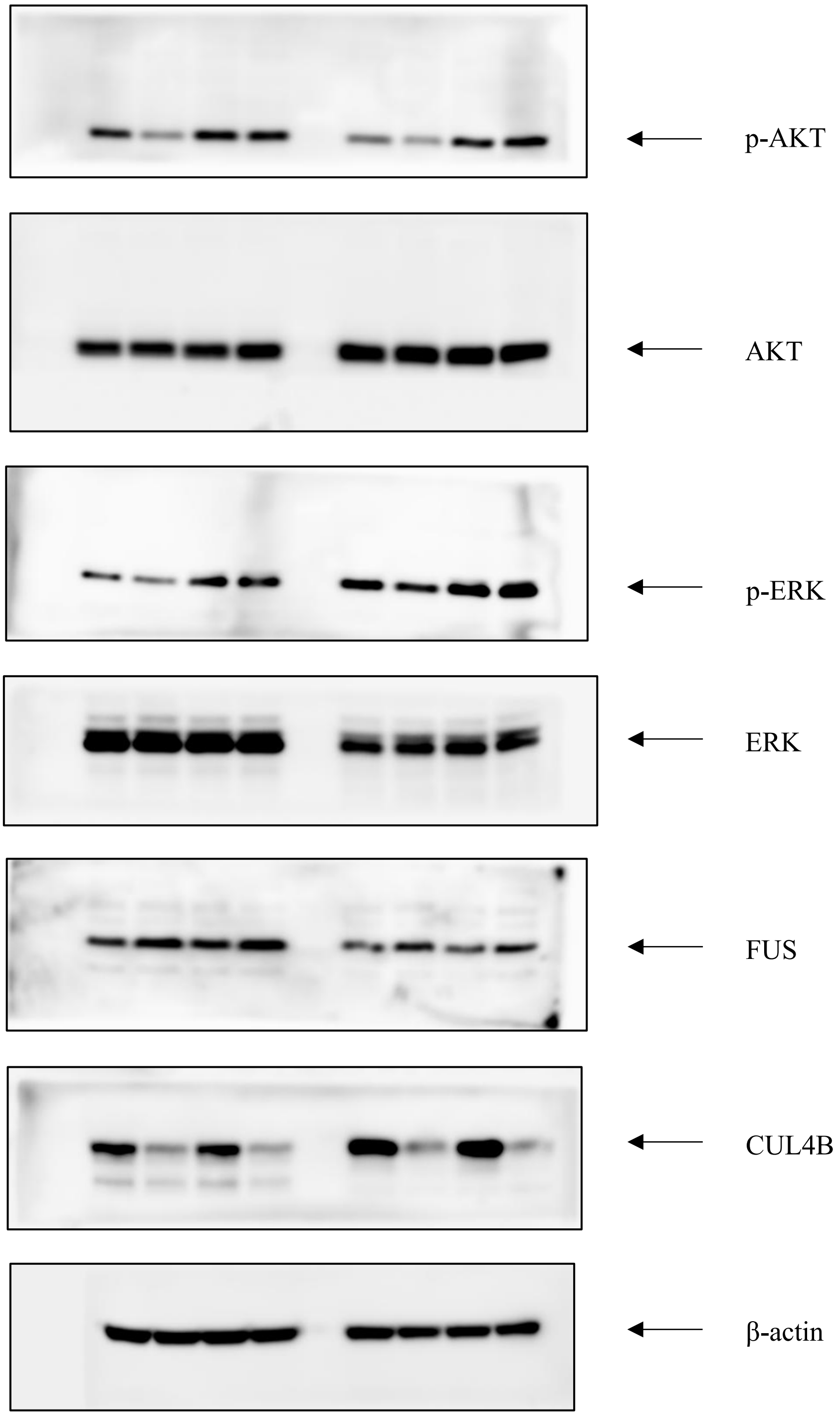

Full unedited blot/gel for Supplemental Figure 2

A.

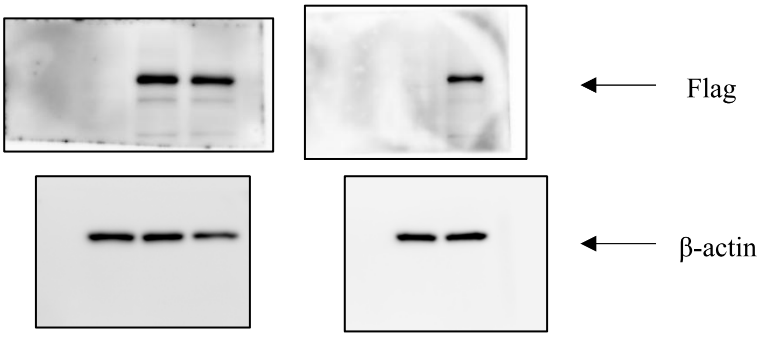

B.

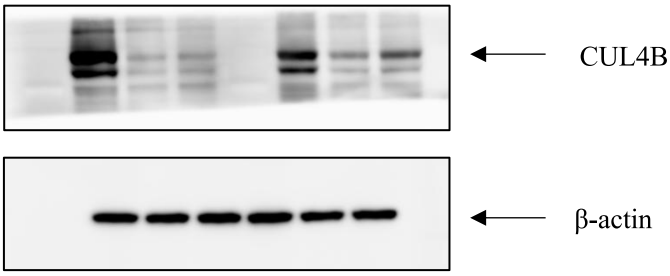

Full unedited blot/gel for Supplemental Figure 4A

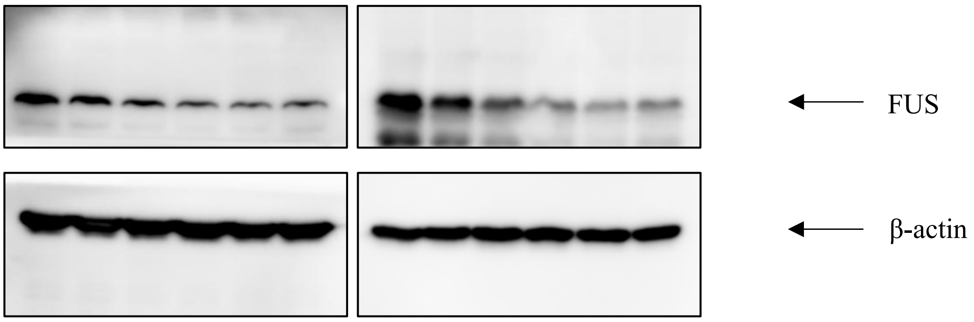

Full unedited blot/gel for Supplemental Figure 4B

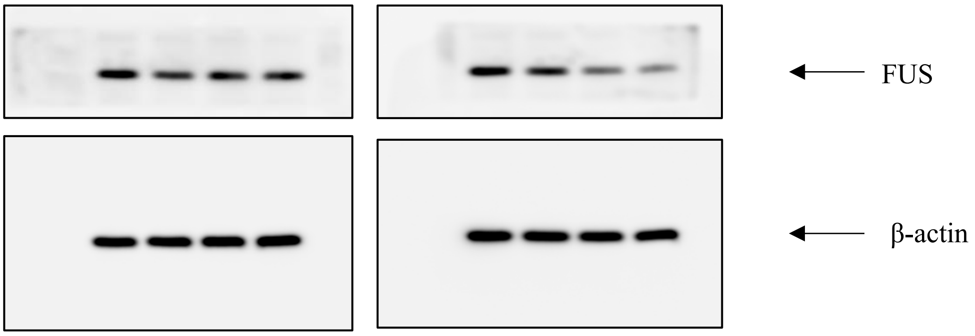

Full unedited blot/gel for Supplemental Figure 4C

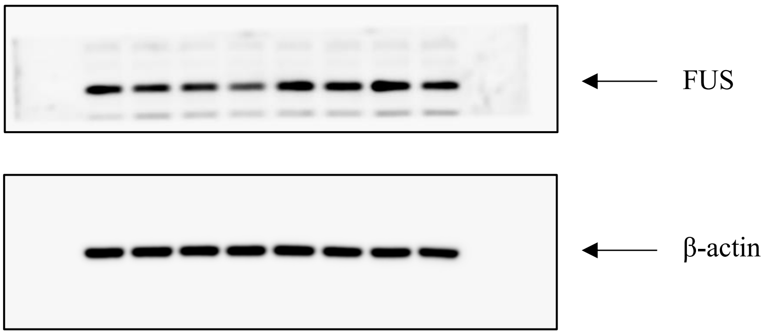

Full unedited blot/gel for Supplemental Figure 4D

Huh7 cells

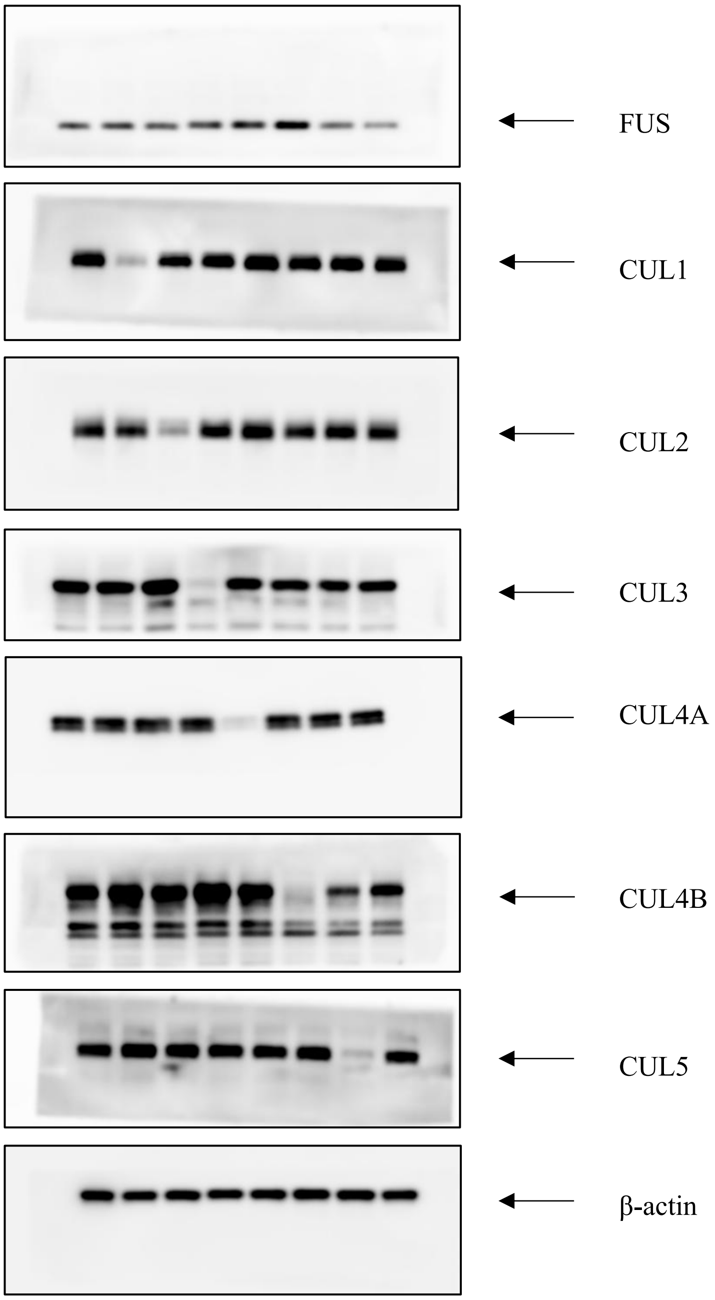

Full unedited blot/gel for Supplemental Figure 4D

LM3 cells

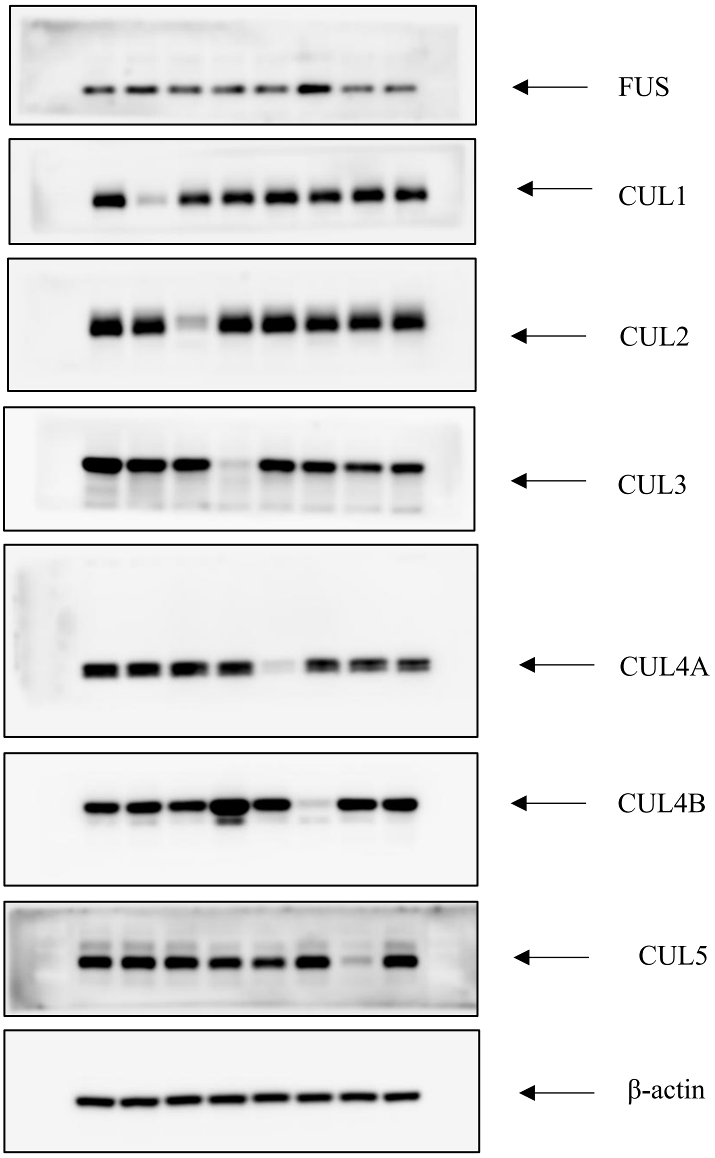

Full unedited blot/gel for Supplemental Figure 5D

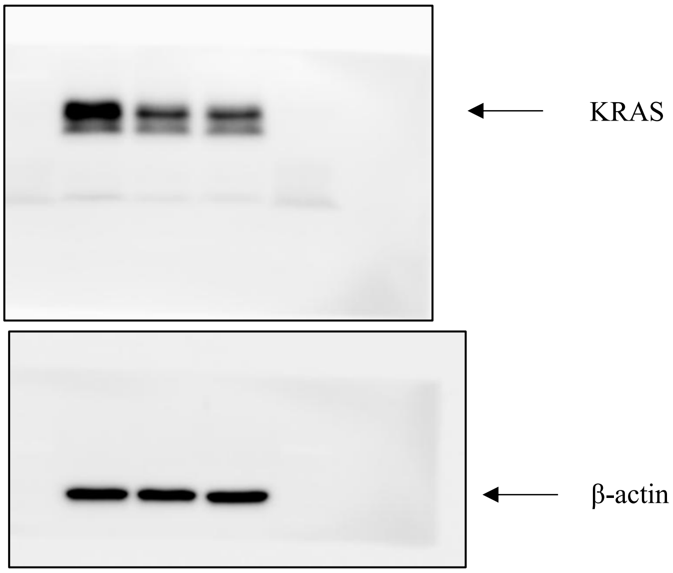

Full unedited blot/gel for Supplemental Figure 6

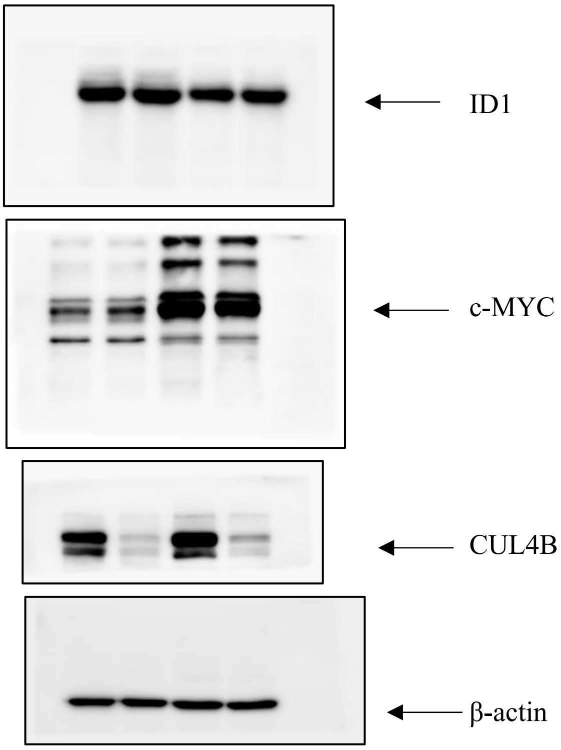

Supplement: Supplementary file 1 — Original Western Blots [file 41419_2025_8320_MOESM1_ESM.pdf]
