# Supplementary material for: CUL4B promotes hepatocellular carcinoma progression and oxaliplatin resistance by facilitating FUS degradation
Source: Cell Death Dis. 2025 Dec 14;17(1):116. doi: 10.1038/s41419-025-08320-6 (PMC12848007; doi:10.1038/s41419-025-08320-6)
Supplement: Supplementary file 2 — Supplemental Figures and Methods [file 41419_2025_8320_MOESM2_ESM.pdf]

Supplemental Figures and Methods

Supplemental Figure 1

Determination of cut-offs by survival ROC curves analysis

Supplemental Figure 2

The efficiency of CUL4B overexpression or knockdown

Supplementary Figure 3

Generation of subcutaneous tumor models

Supplemental Figure 4

FUS is degraded by proteasome

Supplemental Figure 5

KRAS promotes HCC progression and oxaliplatin resistance

Supplementary Figure 6

The effect of CUL4B knockdown on ID1/c-MYC.

Supplemental Table 1

Clinicopathologic parameters according to the expression of CUL4B and miR-143-3p

Supplemental Table 2

Univariate and Multivariate analysis for patient survival with respect to the expression of CUL4B and miR-143-3p

Supplemental Materials and Method

Supplemental Figure 1

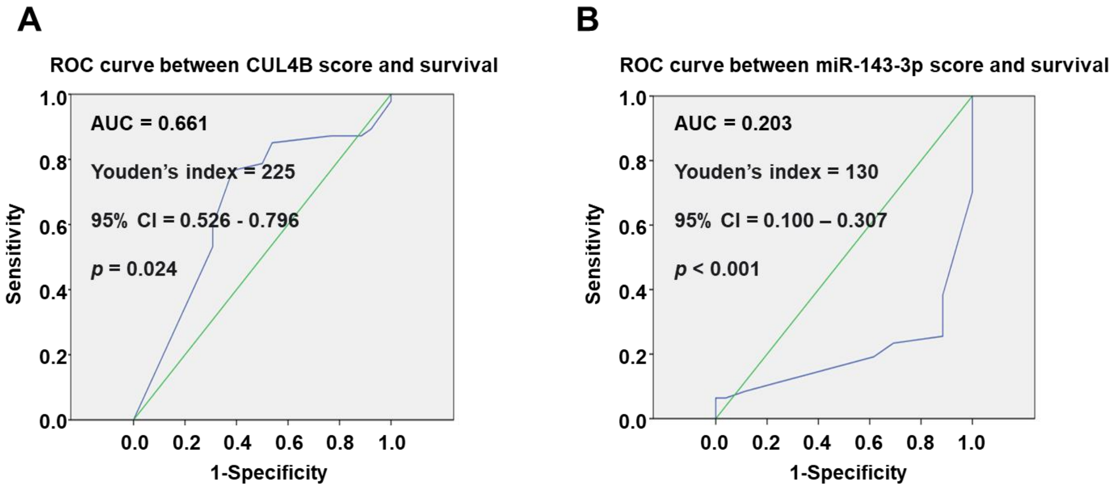

Supplementary Figure 1. Determination of cut-offs by survival ROC curves analysis

Supplemental Figure 2

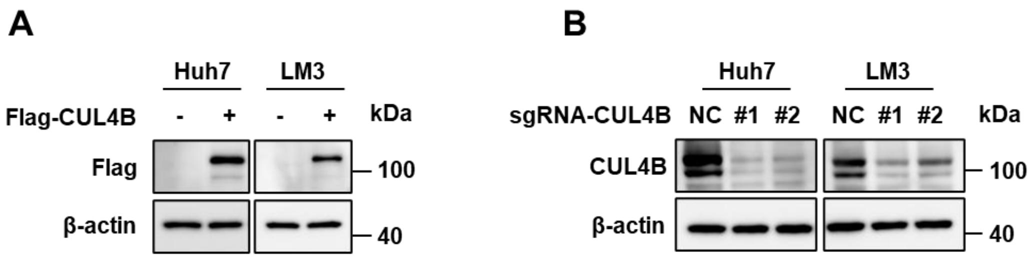

Supplementary Figure 2. The efficiency of CUL4B overexpression or knockdown

Supplemental Figure 3

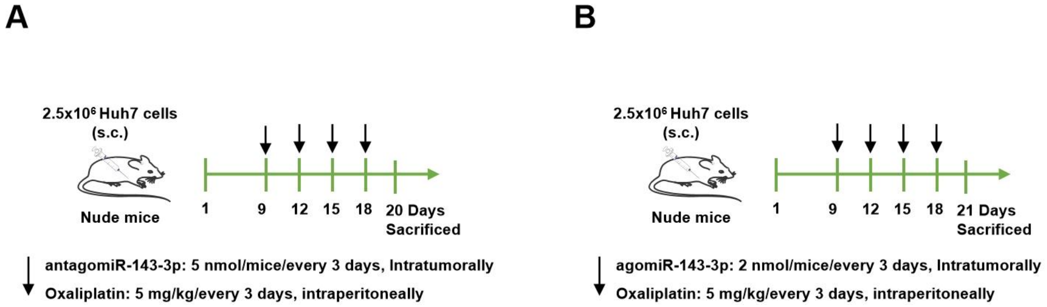

Supplementary Figure 3. Generation of subcutaneous tumor models. A total of 2 × 10^6 stable cells were subcutaneously injected into the right armpits of nude mice (Day 1). Thereafter, the mice were intraperitoneally injected with saline or oxaliplatin (Day 9) (5 mg/kg, once every 3 days) and intratumorally injected with antagomiR-NC or agomiR-143-3p.

Supplemental Figure 4

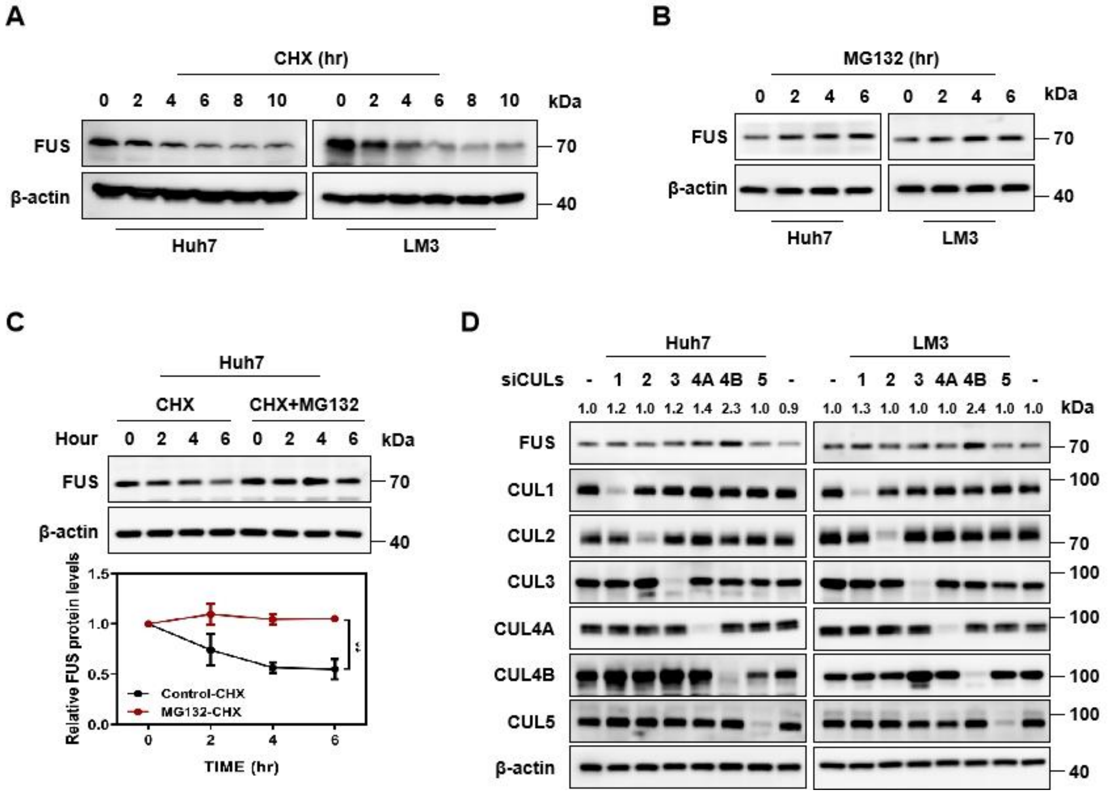

Supplementary Figure 4. FUS is degraded by proteasome. (A) The half-life of FUS was determined by CHX-chase experiments. Cells were treated with 50 μg/mL CHX for 0, 2, 4, 6, 8 or 10 h before being subjected to western blotting analysis. (B) Treatment of proteasome inhibitor MG132 led to an accumulation of FUS. Cells were treated with 10 μM MG132 for 0, 2, 4 or 6 h before being subjected to western blotting analysis. (C) MG132 extended the half-life of FUS. Huh7 cells were treated with 50 μg/mL CHX along with DMSO or MG132 (10 μM) and then harvested for western blotting analysis. FUS protein expression was quantified using ImageQuant TL software, with levels normalized to that of β-actin for comparison (mean ± SD,

n = 3). (D) Knockdown of CUL4B, but not other CULs, led to an accumulation of FUS. Level of FUS expression determined using western blotting analysis following downregulation of CULs in Huh7 and LM3 cells by siRNA interference for 72 h. Two-tailed, unpaired *t* test for **C.**

Supplemental Figure 5

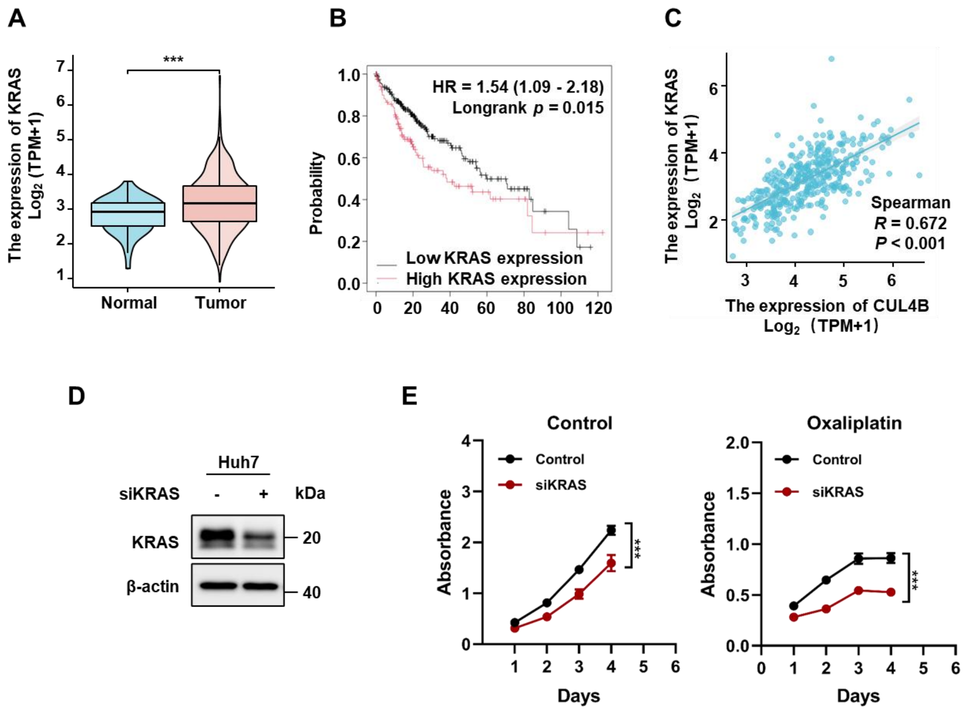

Supplementary Figure 5. KRAS promotes HCC progression and oxaliplatin resistance. (A) Analysis of RNA-sequencing data from a TCGA dataset showed that the mRNA expression of KRAS was higher in HCC tissues than in normal tissues. mRNA transcriptome data in TPM format were obtained from The Cancer Genome Atlas (TCGA) database. KRAS expression analysis was conducted using data from 50 normal tissues and 374 HCC tissues. The Wilcoxon rank sum test was applied to assess differences in expression levels between cancerous and normal tissues. (B) Kaplan-Meier analysis revealed that patients with HCC with higher KRAS expression had lower OS rates than those with lower KRAS expression**.** Kaplan Meier survival curve analysis is performed by the online website Kaplan-Meier plotter

(https://kmplot.com/analysis/). Patients were divided into high group (high) and low group (low) according to the median of KRAS expression. Number at risk refers to the count of individuals who are still survival at that time point. (C) Analysis of RNA sequencing data from TCGA dataset indicated that the mRNA expression of KRAS in HCC tissues was significantly positively correlated with that of CUL4B. The expression correlation of KRAS and CUL4B was analyzed using data from 539 HCC tissues. The Spearman method was used to analyze the correlation. (D) The efficiency of KRAS knockdown. (E) Effect of KRAS knockdown on the proliferation of Huh7 cells treated with or without 2 μM oxaliplatin. Cell proliferation of HCC cells was determined using a CCK8 growth assay for 4 days (mean ± SD, $n = 3$). Two-tailed, unpaired t-test was used.

Supplemental Figure 6

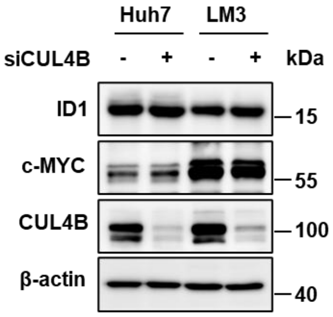

Supplementary Figure 6. The effect of CUL4B knockdown on ID1/c-MYC.

Supplemental Table 1

Clinicopathologic parameters according to the expression of CUL4B and miR-143-3p							
**Variable**	**Overall**	**CUL4B**		***P***	**miR-143-3p**		***P***
	NO. (%)	Low	High		Low	High	
Age				0.568			0.964
<60	56 (76.72)	22 (30.14)	34 (46.58)		30 (41.10)	26 (35.61)	
≥60	17 (23.29)	6 (8.22)	11(15.07)		9 (12.33)	8 (10.96)	
Gender				0.067			0.121
Female	5 (6.85)	4 (5.48)	1 (1.37)		1 (1.37)	4 (5.48)	
Male	68 (93.15)	24 (32.88)	44(60.27)		38 (52.05)	30 (41.10)	
Tumor size				0.020			0.322
≤5	32 (43.84)	18 (21.92)	14(21.92)		15 (20.54)	17 (23.29)	
>5	41 (56.17)	12 (16.44)	29 (39.73)		24 (32.88)	17 (23.29)	
Tumor stage				0.037			0.043
I+II	38 (52.06)	20 (27.40)	18 (24.66)		16 (21.91)	22 (30.14)	
III+IV	35 (47.95)	10 (13.70)	25 (34.25)		23 (31.51)	12 (16.44)	

Supplemental Table 2

Univariate and Multivariate analysis for patient survival with respect to the expression of CUL4B and miR-143-3p

Variable	Univariable	*p*	Multivariable	*p*
	HR (95% CI)		HR (95% CI)	
**Age: ≥60 *vs.* <60 (year)**	0.758 (0.376-1.527)	0.438	0.583 (0.271-1.256)	0.168
**Gender: female *vs.* male**	0.460 (0.111-1.906)	0.285	0.657 (0.157-2.753)	0.565
**Tumor size: >3 *vs.* ≤3**	1.940 (1.063-3.540)	0.031	0.732 (0.322-1.663)	0.456
**TNM stage: III+IV *vs.* I+II**	3.662 (1.953-6.867)	<0.001	3.310 (1.437-7.628)	0.005
**CUL4B: high *vs.* low**	2.755 (1.447-5.246)	0.002	1.980 (1.012-3.875)	0.046
**miR-143-3p: high *vs.* low**	0.225 (0.115-0.438)	<0.001	0.257 (0.129-0.513)	<0.001

Supplemental Materials and Methods

miRNAs sequencing analysis

RNA sequencing analysis for this study was conducted by LC-Bio Technologies (Hangzhou, China). Total RNA was extracted from cells treated with DMSO or MLN4924 using TRIzol reagent (Invitrogen) following the manufacturer's protocol. A small RNA library was constructed using the TruSeq Small RNA Sample Prep Kits (Illumina, San Diego, USA). Adapter-ligated RNA was reverse transcribed into cDNA, followed by polymerase chain reaction (PCR) amplification. The resulting PCR products were purified to create small RNA libraries. Single-end sequencing (1 × 50 bp) was performed on the Illumina Hiseq2500 platform (Illumina). The miRNA sequencing data were analyzed using ACGT101-miR (LC Sciences, Houston, Texas, USA). The analysis process included the following steps: (1) Removal of the 3' adapter and low-quality sequences to generate clean data; (2) Length screening to retain sequences between 18-26 nt; (3) Filtering through comparison with various RNA databases, including mRNA, RFam, and Repbase; (4) Identification of miRNAs by comparison with precursor and genome sequences; (5) Differential analysis of miRNAs. Differentially expressed miRNAs were identified based on the criteria of fold change ≥ 2 and p-value ≤ 0.05. Kyoto Encyclopedia of Genes and Genomes (KEGG) pathway analysis and Gene Ontology (GO) analyses were performed using the online platform OmicStudio (www.omicstudio.cn).

miRNA mimic/miRNA inhibitors/siRNA transfection

Cells were transfected with miRNA mimics, miRNA inhibitors, or siRNA oligonucleotides using Lipofectamine RNAiMAX (Invitrogen, USA). At the time of transfection, cells were seeded to reach 60% confluency (using a 6-well plate as an example). For the transfection mix, 3 μL of Lipofectamine RNAiMAX Reagent and 3 μL of 20 μM oligonucleotides were each diluted in 200 μL of Opti-MEM medium (Gibco, 31985070). The diluted oligonucleotides were then combined with the diluted Lipofectamine RNAiMAX Reagent, and after a 15-minute incubation period, the oligonucleotide-lipid complex was added to the cells. Cells were harvested 72 hours post-transfection. The siRNA oligonucleotides were synthesized by GenePharma (Shanghai, China), while the miR-143-3p mimic and inhibitor oligonucleotides were synthesized by Sangon Biotech (Shanghai, China). The sequences are as follows:

Nontarget control (NC): 5′-UUCUCCGAACGUGUCACGU-3′;

si*CUL1*: 5'-CUAGAUACAAGAUUAUACAUGCGG-3';

si*CUL2*: 5'-GCACAAUGCCCUUAUUCAA-3';

si*CUL3*: 5'-UUGACGUGAACUGACAUCCACAUUC-3';

si*CUL4A*: 5'-GAAGAUUAACACGUGCUGG-3';

si*CUL4B*: 5'-AAGCCUAAAUUACCAGAAA-3';

si*CUL5*: 5'-CUACUGACUCUGAGAAAUA-3';

si*DDB1*: 5'- CCUAUCACAAUGGUGACAAAU-3';

si*RBX1*: 5'- GACUUUCCCUGCUGUUACCUAA-3';

si*FUS*-1: 5'-CGUGGUGGCUUCAAUAAAU-3';

si*FUS*-2: 5'-CAGCCCAUGAUUAAUUUGU-3';

si*KRAS*: 5'-GAGGAGUACAGUGCAAUGA-3';

NC-mimic Sense: UUGUACUACACAAAAGUACUG;

NC-mimic Antisense: GUACUUUUGUGUAGUACAAUU;

miR-143-3p-mimic Sense: UGAGAUGAAGCACUGUAGCUC;

miR-143-3p-mimic Antisense: GCUACAGUGCUUCAUCUCAUU;

NC-inhibitor: CAGUACUUUUGUGUAGUACAA (modified by methoxy);

miR-143-3p-inhibitor: GAGCUACAGUGCUUCAUCUCA (modified by methoxy).

Real-time reverse transcription polymerase chain reaction for mRNA

Total RNA was extracted from cells using an RNA purification kit (EZBioscience, B004D). Reverse transcription was performed on 1 μg of total RNA per sample using the PrimeScript™ RT Reagent Kit (TaKaRa, RR047A). Real-time polymerase chain reaction (RT-PCR) was subsequently conducted using TB Green® Premix Ex Taq™ (TaKaRa, RR420A) on the ABI StepOne Plus thermocycler (Thermo Fisher Scientific, USA), following the manufacturer's instructions. Data were analyzed using the $2^{-\Delta\Delta Ct}$ method, with β-actin serving as the endogenous control

for normalization. The primers for PCR were synthesized by Sangon Biotech (Shanghai, China), and their sequences were as follows:

β-actin forward: TCCCTGGAGAAGAGCTACG;

β-actin reverse: GTAGTTTCGTGGATGCCACA;

KRAS forward: ACAGAGAGTGGAGGATGCTTT;

KRAS reverse: TTTCACACAGCCAGGAGTCTT;

Real-time reverse transcription polymerase chain reaction for miR-143-3p

RNA was extracted from cells using the Universal MicroRNA Purification Kit (EZBioscience, EZB-miRN1). Reverse transcription was carried out on 1 μg of RNA per sample using the PrimeScript™ RT Reagent Kit (TaKaRa, RR047A). The expression levels of miR-143-3p were quantified by RT-PCR using TB Green® Premix Ex Taq™ (TaKaRa, RR420A) on the ABI StepOne Plus thermocycler (Thermo Fisher Scientific, USA), following the manufacturer's instructions. Relative miRNA expression was normalized to U6 using the $2^{-\Delta\Delta Ct}$ method. The primers for PCR were synthesized by Sangon Biotech (Shanghai, China), and their sequences were as follows:

U6 forward: CTCGCTTCGGCAGCACA;

U6 reverse: AACGCTTCACGAATTTGCGT;

miR-143-3p RT primer:

GTCGTATCCAGTGCAGGGTCCGAGGTATTCGCACTGGATACGACGAGCTA;

miR-143-3p forward: ATGCGCTGAGATGAAGCACT;

miR-143-3p reverse: CAGTGCAGGGTCCGAGGT.

Immunoprecipitation and Western blotting

For anti-Flag immunoprecipitation, Huh7 cells were lysed in ice-cold RIPA buffer (Beyotime, P0013D) with added protease inhibitors (Beyotime, P1005 and ST507). The lysates were clarified by centrifugation at 12,000 rpm for 15 minutes, and the supernatants were incubated with anti-Flag M2 affinity resin (Sigma-Aldrich, A2220) at 4°C for 3 hours with rotation. The resin was then washed three times with ice-cold RIPA buffer and the bound proteins were analyzed by SDS-PAGE followed by western blotting.

Western blotting was performed using standard protocols. Cells or immunoprecipitated products were mixed with SDS-PAGE sample loading buffer (Beyotime, P0015A), heated at 100°C for 10 minutes, and clarified by centrifugation. Equal amounts of protein were separated by SDS-PAGE and transferred to 0.2 μm polyvinylidene difluoride membranes (Millipore, ISEQ00010). Membranes were blocked with 5% nonfat milk in TBST (TBS with 0.1% Tween 20) at room temperature for 1 hour, then incubated with primary antibodies overnight at 4°C. After washing, secondary antibodies (anti-mouse: Proteintech, RGAM001, 1:3000 dilution; anti-rabbit: BIOKE, BK-R05, 1:3000 dilution) were applied and incubated at room temperature for 1 hour. Following three washes with TBST, signals were developed using a Western ECL kit (Epizyme, SQ201) and images were captured with an

Amersham Imager 680 (GE Healthcare, USA). Protein expression was quantified using ImageQuant TL software, with levels normalized to β-actin for comparison.

Analysis of human HCC tissue arrays

A tissue microarray containing human HCC and adjacent normal tissues was purchased from Shanghai Outdo Biotech Company (Shanghai, China). This study was approved by the Ethics Committee of Shanghai Outdo Biotech Company. All patients provided written informed consent prior to their inclusion in the study.

For immunohistochemical analysis, tissue sections from the human HCC array were deparaffinized and treated with 3% hydrogen peroxide to block endogenous peroxidase activity. Antigen retrieval was performed using 0.1 M sodium citrate buffer (pH 6.0) for one hour. The sections were then incubated with an anti-CUL4B antibody (Proteintech, 12916-1-AP, 1:3000 dilution) overnight at 4°C. After washing, the sections were treated with a horseradish peroxidase-conjugated secondary antibody, followed by diaminobenzidine substrate for signal detection. The sections were counterstained with hematoxylin, dehydrated, and examined using an Aperio ScanScope XT photomicroscope (Leica Microsystems, Inc.).

For in situ hybridization of miR-143-3p, probes and kits were designed and synthesized by Wuhan Boster Company (MK10112). The 3' end of the probe was labeled with digoxin. Briefly, paraffin-embedded tissue sections were deparaffinized, rehydrated, treated with 3% H_2O_2 for 10 minutes at room temperature, digested with pepsin in 3% citric acid at 37°C for 10 minutes, and post-fixed with 1%

paraformaldehyde at room temperature for 10 minutes. The sections were then hybridized overnight with a miR-143-3p-specific probe at 42°C, followed by incubation with a biotinylated anti-digoxin antibody at 37°C for 60 minutes. Streptavidin-Biotin Complex (SABC) was applied at 37°C for 20 minutes, followed by incubation with biotinylated peroxidase at 37°C for 20 minutes. The signal was visualized by staining with DAB and counterstaining with hematoxylin, then examined using the Aperio ScanScope XT photomicroscope (Leica Microsystems, Inc.).

For evaluation, tissue sections were semi-quantitatively assessed based on the percentage of positively stained tumor cells and the intensity of staining. Staining intensity was categorized into four grades: no staining = 0, weak staining = 1, moderate staining = 2, and strong staining = 3. The percentage of positive cells was calculated and scored from 0 to 100%. The proportion and intensity scores were multiplied to yield a total score ranging from 0 to 300 (0: 0% of cells stained; 300: 100% of cells with strong staining intensity).

Molecular dynamics simulations

This experiment used GROMACS (2023-4 version) to simulate proteins in a cubic box with SPC water molecules and 0.15 M sodium chloride at 310 K and 1 atm. The simulation ran for 100 ns, capturing trajectory data every 1 ns. The stability of FUS and $CRL4B^{DTL}$ complex were assessed by calculating RMSD values of the backbone α-carbon atoms, which initially increased but stabilized around 50 ns at

0.6-0.7 nm, indicating a stable state with minor structural changes. The flexibility was evaluated by calculating RMSF values of each residue, showing most regions were stable with low RMSF values, while some regions had RMSF values above 1.0 nm, indicating higher flexibility or potential functional activity. The analysis of Rg showed relative stability without significant fluctuations, indicating the protein remained in a thermodynamic equilibrium state without substantial conformational changes with Rg values of 100 ns.

Analysis of RNA-sequencing data from a TCGA dataset

mRNA transcriptome data in TPM format were obtained from The Cancer Genome Atlas (TCGA) database. KRAS expression analysis was conducted using data from 50 normal tissues and 374 HCC tissues. The Wilcoxon rank sum test was applied to assess differences in expression levels between cancerous and normal tissues.

Kaplan-Meier survival analysis

Kaplan Meier survival curve analysis is performed by the online website Kaplan-Meier plotter (https://kmplot.com/analysis/). HCC mRNA data set was selected for analysis. Patients were divided into high group (high) and low group (low) according to the median of KRAS expression. Number at risk refers to the count of individuals who are still survival at that time point.
